# Engineered Mesenchymal Stem Cell‐Derived Extracellular Vesicles Scavenge Self‐Antigens for Psoriasis Therapy via Modulating Metabolic and Immunological Disorders

**DOI:** 10.1002/advs.202410067

**Published:** 2024-12-12

**Authors:** Xin Zhou, Bo Tang, Qing Huang, Siyu Yang, Yang Jiang, Lizhou Xu, Wen Chen, Guangchang Shan, Xuankai Liao, Chongchao Hou, Zhihong Yao, Chaowei Zou, Rongying Ou, Yunsheng Xu, Danyang Li

**Affiliations:** ^1^ Research Center The Seventh Affiliated Hospital Sun Yat‐sen University Shenzhen 518107 China; ^2^ Department of Dermatovenereology The Seventh Affiliated Hospital Sun Yat‐sen University Shenzhen 518107 China; ^3^ Department of Pathology The Affiliated Traditional Chinese Medicine Hospital Southwest Medical University Luzhou 646000 China; ^4^ ZJU‐Hangzhou Global Scientific and Technological Innovation Center Zhejiang University Hangzhou 311215 China; ^5^ Department of Pathology The Seventh Affiliated Hospital Sun Yat‐sen University Shenzhen 518107 China; ^6^ Shenzhen Wingor Biotechnology Co., ltd Shenzhen 518107 China; ^7^ Department of Clinical Medicine Zhongshan Medical School Sun Yat‐sen University Guangzhou 528478 China; ^8^ Department of Gynaecology and Obstetrics The First Affiliated Hospital Wenzhou Medical University Wenzhou 325000 China

**Keywords:** arginase1, mesenchymal stem cell‐derived extracellular vesicles, metabolic and immunological modulation, psoriasis therapy, self‐antigens

## Abstract

Psoriasis is a chronic inflammatory dermatosis driven by excessive activation of the immune system. Recent studies have demonstrated the therapeutic potential of mesenchymal stem cell‐derived extracellular vesicles (MSC‐EVs) to psoriasis because of their immunomodulation functions. Yet, the outcome of MSC‐EVs alone is still unsatisfactory and the underlying mechanisms are also unclear. Here, it is uncovered that arginase1 (Arg1)/polyamine is overexpressed in psoriasis patients and murine, inducing the in‐situ accumulation of self‐antigens. Engineered nor@MSC‐EVs are fabricated by loading Arg1 inhibitor nor‐NOHA into MSC‐EVs for studying the therapeutic effect and mechanism of psoriasis. The nor@MSC‐EVs exhibited profound suppression of the NF‐κB signaling pathway by targeting Arg1/polyamine‐mediated DCs/Th17 axis through scavenging self‐antigens, resulting in superior mitigation of skin lesions and modulation of local and systemic metabolic and immunological disorders compared to the MSC‐EVs and clinically used anti‐IL17A both in vitro and in vivo. Together, the results highlight a novel perspective for psoriasis therapy by nor@MSC‐EVs with broad clinical translational potential.

## Graphical abstract

An engineered nor@MSC‐EVs are fabricated by encapsulating arginase1 inhibitor nor‐NOHA into MSC‐EVs. The nor@MSC‐EVs exhibited profound suppression of the NF‐κB signaling pathway by targeting Arg1/polyamine‐mediated DCs/Th17 axis through scavenging self‐antigens, resulting in superior mitigation of skin lesions and modulation of local and systemic metabolic and immunological disorders.

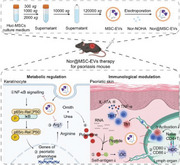

## 1 Introduction

Psoriasis is a chronic inflammatory dermatosis characterized by erythematous plaques and silvery scales.^[^ ^1^ ^]^ It is primarily driven by excessive activation of both the innate and adaptive immune systems. Particularly, psoriasis‐specific self‐antigens presented by dendritic cells (DCs) initiate the activation and clonal expansion of antigen‐specific CD4+ T cells, which subsequently secrete excessive cytokines. This dysregulation of the IL23/Th17 axis ultimately leads to hyperproliferation and inflammation of the epidermis.^[^ ^2^ ^]^

Traditional therapeutic approaches, such as methotrexate and cyclosporine, have been utilized to manage psoriasis by inhibiting systemic immunity since the 1980s.^[^ ^3^ ^]^ However, their applications are hindered due to the associated risks, including infections and malignancies.^[^ ^1^, ^4^ ^]^ Recently developed bioagents targeting key cytokines involved in psoriasis pathogenesis, such as IL‐17A, and IL‐23, have shown efficacy in alleviating psoriatic symptoms.^[^ ^5^ ^]^ Despite their benefits, completely curing the disease by merely neutralizing cytokines from the overactive immune system remains challenging.^[^ ^6^ ^]^

Mesenchymal stem cells (MSCs) and their derived extracellular vesicles (EVs) have emerged as novel strategies for psoriasis treatment due to their remarkable immunomodulatory and anti‐inflammatory properties.^[^ ^7^ ^]^ A phase 1/2a clinical trial reported that MSC treatment resulted in a 40% improvement in 47% of the patients.^[^ ^7^, ^8^ ^]^ Similarly, recent studies have demonstrated that MSC‐EVs injections reduce skin inflammation in psoriasis mice model by regulating cytokines expression.^[^ ^9^ ^]^ Indeed, MSC‐EVs have shown significant promise in pre‐clinical studies of other autoimmune diseases,^[^ ^10^ ^]^ highlighting their potential in psoriasis treatment. Nevertheless, recent studies have also identified metabolic pathways as crucial contributors to the epithelial and immune mechanisms underlying psoriasis.^[^ ^11^ ^]^ Lou et al. revealed that the RNA+polyamine+Hnrnpa_1226‐237_ complex^[^ ^12^ ^]^ is considered as a self‐antigen, with aberrant polyamine metabolism driven by the overexpression of arginase‐1 (Arg1) in psoriatic keratinocytes enhancing the sensing of this complex by DCs.^[^ ^13^ ^]^ Therefore, targeting metabolic and immunological disorders via eliminating psoriasis‐related self‐antigens could offer a synergistic strategy for psoriasis therapy.^[^ ^14^ ^]^

**Scheme 1:**
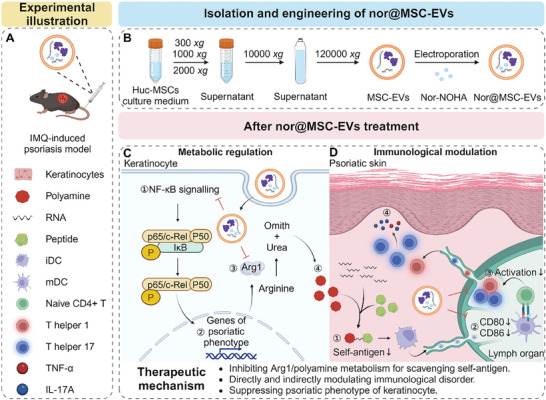
Schematic illustration of nor@MSC‐EVs for synergistic therapy against psoriasis by modulating both local and systemic metabolic and immunological disorders.

Here, we reported a novel engineered MSC‐EVs (nor@MSC‐EVs), which are loaded with Arg1 inhibitor (nor‐NOHA) through electroporation for the synergistic therapy of psoriasis via modulating both local and systemic metabolic and immunological disorders (**Scheme** **1**). We initially investigated the specific overexpression of Arg1 in psoriasis by examining patient samples with varied scaly erythematous skin diseases. Upon intravenous administration in psoriasis mice, nor@MSC‐EVs outperformed MSC‐EVs, nor‐NOHA, and the clinical biological agent anti‐IL17A in decreasing polyamines, inhibiting DC maturation via reducing self‐antigen generation, Th1/17 cells activation, and keratinocyte inflammation, thereby modulating both local and systemic metabolic and immunological disorders. We believe this study provided a prospective translational practice for psoriasis treatment with nor@MSC‐EVs.

## 2 Results

### 2.1 Arg1/Polyamine Mediated the Development of Psoriasis

**Figure 1:**
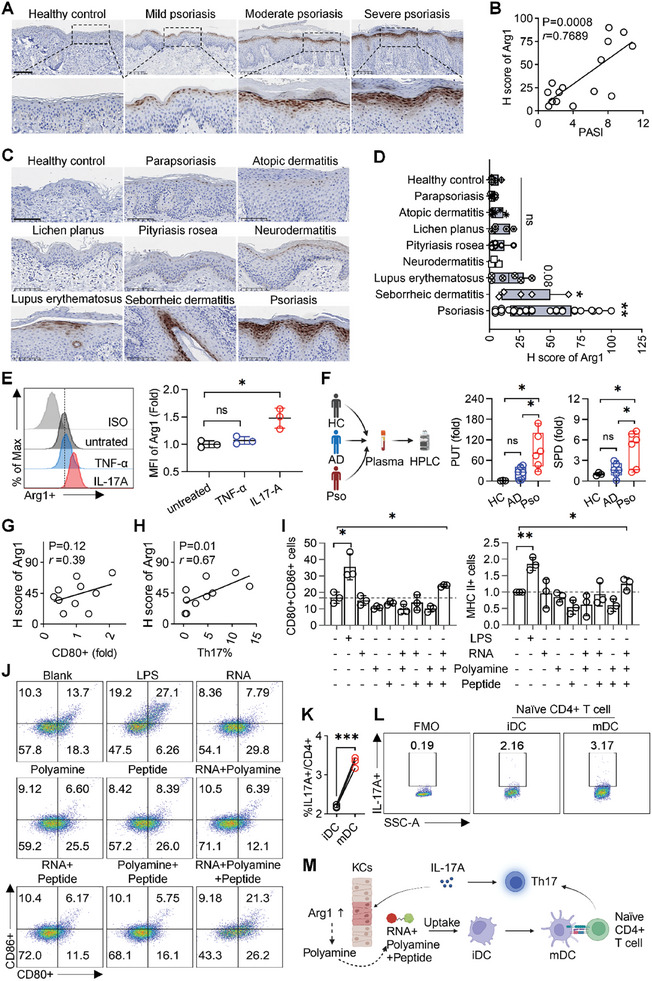
Arg1/polyamine mediated the development of psoriasis. A) IHC staining depicting Arg1 expression in skin sections from healthy controls and psoriasis patients. Scale bar = 200 µm. B) Linear regression analysis demonstrating the correlation between Arg1 expression levels (H score) and the severity of psoriasis (PASI score), n = 16. C) IHC staining depicting Arg1 expression in skin sections from patients with a range of erythematous scaly skin diseases. Scale bar = 200 µm. D) Quantification of Arg1 expression (H score) of different erythematous scaly skin diseases (n = 3 for the neurodermatitis group, n = 28 for psoriasis group, and n = 5 for other groups). E) Flow cytometry analysis of Arg1 expression in HaCaT cells treated with TNF‐α or IL‐17A in vitro (n = 3). F) Analysis of polyamine (putrescine and spermidine) levels in plasma from healthy controls (HC), atopic dermatitis (AD), and psoriasis (Pso) patients (n = 3 for HC, n = 6 for AD and Pso). G) and H) Linear regression analysis of the H score of Arg1 of mouse skin (IHC staining) with immunological markers of mouse skin (flow cytometry): MFI of CD80+ in DCs (G) and Th17% cells (H) (n = 9). I) Statistical analysis of CD80+CD86+ cells and MHC II+ cells in BMDCs (n = 3). J) Representative flow cytometry of CD80+CD86+ cells in BMDCs treated with indicated reagents. K) Statistical analysis and L) representative flow cytometry of the percentage of CD4+IL‐17A+ cells in co‐cultured naïve CD4+ T cells and immature/mature BMDCs (n = 3). M) Schematic diagram depicting the mechanism of Arg1/polyamine mediated development of psoriasis. All data are expressed as mean ± S.D. (n≥3). Linear regression analysis was performed (B, G, and H). Statistical significance was calculated via one‐way ANOVA with a Tukey's test (D‐F, and I) or unpaired two‐tailed t test (K). ns, not significant; *p < 0.05, **p < 0.01, ***p < 0.001.

The key enzymes of amino acid metabolism pathways are pivotal checkpoints involved in regulating autoimmune disease. To investigate the association between Arg1 and psoriasis, Arg1 expression in skin sections from psoriasis patients with different severities was determined via immunohistochemical staining (IHC). Consistent with previous reports,^[^ ^15^ ^]^ we identified that Arg1 was overexpressed in the epidermis of psoriasis patients than healthy controls (**Figure** **1**A). Linear regression analysis demonstrated a positive correlation between Arg1 expression (H score) and with psoriasis area and severity index (PASI score) (Figure 1B), implying that Arg1 was involved in the psoriasis pathogenesis. Additionally, an immunohistochemical analysis of Arg1 on skin sections from patients with scaly erythematous skin diseases similar to psoriasis was conducted. Unlike psoriasis, conditions such as parapsoriasis, atopic dermatitis, lichen planus, pityriasis rosea, and neurodermatitis have shown negligible Arg1 expression despite epidermis hyperplasia (Figure 1C,D). In contrast, Arg1 overexpression was observed in lupus erythematosus, seborrheic dermatitis, and psoriasis due to the varying degrees of involvement in dysregulation of Th17/IL‐17A^[^ ^13^, ^16^ ^]^ (Figure 1C,D). For further validation, an in vitro psoriatic keratinocytes model was established by stimulating HaCaT cells with different cytokines and subsequently the expression of Arg1 was analyzed. It was found that only IL‐17A was able to drive Arg1 up‐regulation (Figure 1E) and increasing Arg1 expression exhibited a concentration‐dependent manner of IL‐17A (Figure S1, Supporting Information). It was known that Arg1 catalyzes arginine hydrolysis to direct products (urea and ornithine) and end products (polyamine). Thus, the level of plasma polyamine (putrescine, PUT and spermidine, SPD) in different groups was examined by High‐Performance Liquid Chromatography (HPLC). It revealed that the PUT and SPD were higher in plasma from psoriasis patients compared to atopic dermatitis patients, while no robust difference was observed between atopic dermatitis and healthy control (Figure 1F), demonstrating the overexpression of Arg1 was psoriasis‐specific and could lead to the perturbations of polyamine metabolism.

We further verified the above findings in an imiquimod (IMQ)‐induced psoriasis mouse model. A linear regression analysis (Figure 1G,H) was performed for the skin samples between the expression of Arg1 by IHC (H score) and immunological markers (CD80 in DCs, and Th17 in CD4+ T cells) via flow cytometry. Positive correlations were identified between CD80 (Figure 1G), Th17 (Figure 1H) and Arg1 H score. Together, these data demonstrated an inner link between Arg1 and innate immune cells (DCs) and adaptive immune cells (Th17), which might be IL‐17A secreted by DC/Th17A axis driving the Arg1 upregulation in psoriasis.^[^ ^3^, ^13^, ^16^ ^]^

Nevertheless, the effect of Arg1 and its metabolites (such as urea and polyamines) on immune cells of psoriasis was unclear. Previous studies have shown that the RNA from psoriatic keratinocytes complexed with peptide could activate monocyte‐derived DCs.^[^ ^17^ ^]^ The maturation of DCs subsequent activation of DCs/Th17 axis indicated there was a loop of metabolic and immunological disorders mediated by Arg1/polyamine. To this, we investigated the impact of Arg1/polyamine on the DCs/Th17 axis by assessing the maturation markers of bone marrow derived DCs (BMDCs). Stimulation with RNA+polyamine+peptide complex significantly increased the maturation markers of BMDCs compared to other single or combination stimulators (Figure 1I,J), emphasizing the role of this complex in immunological disorder modulation. Co‐culturing naïve CD4+ T cells with BMDCs pre‐treated with or without RNA+polyamine+peptide complex revealed that mature DCs (mDCs) promoted the differentiation of CD4 + IL17A + cells (Th17) (Figure 1K,L). In summary, the above results demonstrated the psoriasis‐specific overexpression of Arg1 and its contribution to the cyclic relationship between metabolic and immunological disorders mediated by Arg1/polyamine, as depicted in Figure 1M.

### 2.2 Reducing Psoriasis‐Specific Self‐Antigens Decreased the Maturation of DCs and Activation of Th17 Cells

**Figure 2:**
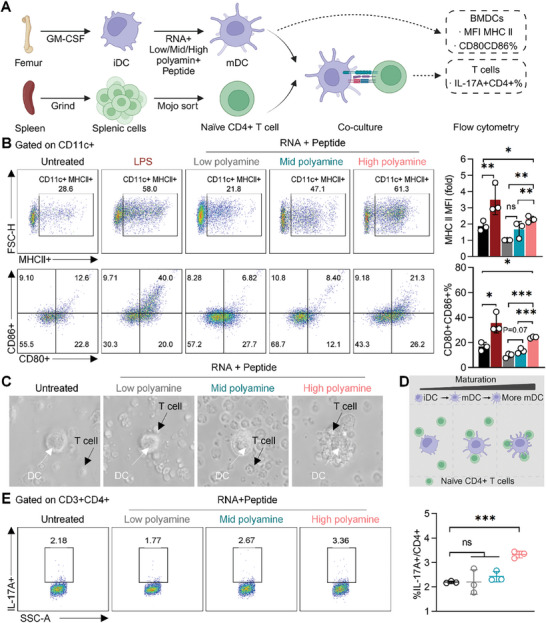
Reducing psoriasis‐specific self‐antigens decreased the maturation of DCs and activation of Th17 cells. A) Schematic diagram of co‐culture of naïve CD4+ T cells and BMDCs pre‐treated with psoriasis‐specific antigens (RNA+low/mid/high+polyamine+peptide complex). B) Flow cytometry analysis of MHC II and CD80+CD86+ expression in BMDCs treated with PBS, LPS, RNA+low/mid/high polyamine+peptide complex. C,D) Bright field microscope images (C) and schematic diagram (D) of co‐culture of naïve CD4+ T cells and BMDCs with different degrees of maturation. E) Flow cytometry analysis of IL17A+CD4+ T cells activated by BMDCs pre‐treated with RNA+low/mid/high polyamine+peptide complex. All data are expressed as mean ± S.D. (n = 3). Statistical significance was calculated via one‐way ANOVA with a Tukey's test. ns, not significant; *p < 0.05, **p < 0.01, ***p < 0.001.

Self‐antigens can bind to Toll‐like receptors on the surface of DCs in the dermis of patients with psoriasis, triggering DCs activation and subsequent clonal expansion of antigen‐specific CD4+ T cells.^[^ ^3^ ^]^ To investigate the impact of decreasing self‐antigens on BMDCs maturation, we incubated BMDCs with RNA+low/mid/high polyamine+peptide before the assessment of the maturation markers (**Figure** **2**A). Stimulated by RNA+polyamine+peptide complex, robust elevations of maturation markers expression were observed with the increasing of polyamine concentration. Notably, the RNA+high polyamine+peptide complex exhibited the highest increase in MHC II expression (Figure 2B), and elevation of CD80 and CD86 (Figure 2B), compared to other lower polyamine concentration groups, demonstrating the reduced polyamine concentration correlated with diminished maturation state of BMDCs.

We then co‐cultured the above BMDCs and naïve CD4+ T cells for 72 h (Figure 2A). Microscope images revealed decreased interactions between CD4+ T cells and BMDCs due to the reduced MHC II expression on DCs, leading to diminished cell‐cell interaction (Figure 2C,D).^[^ ^18^ ^]^ Flow cytometry analysis also demonstrated a decrease in IL17A+CD4+ T cells percentage when co‐cultured with BMDCs pre‐treated with RNA+low/mid polyamine+peptide complex (1.77% and 2.67% respectively) compared to the high polyamine group (3.36%) (Figure 2E), indicating that reducing polyamine concentration can attenuate DC‐T cell interactions and lead to lower Th17 differentiation. Together, targeting Arg1‐mediated polyamine production provides a novel strategy to interfere the DCs/Th17 axis and may offer therapeutic benefits for psoriasis therapy.

### 2.3 Engineering and Characterizations of nor@MSC‐EVs

To address the metabolic and immunological disorder of psoriasis, we developed MSC‐EVs loaded with nor‐NOHA, an Arg1 inhibitor. First, human umbilical cords MSCs were isolated and cultured, demonstrating fibroblast‐like morphology (Figure S2A, Supporting Information). Their stemness was further verified by Oil Red O and Alizarin Red S staining after osteogenesis and adipogenesis differentiation (Figure S2B, Supporting Information). Flow cytometry analysis validated the MSC identity by both the positive (CD73 and CD105) and negative markers (CD34 and CD45) (Figure S2C, Supporting Information).

**Figure 3:**
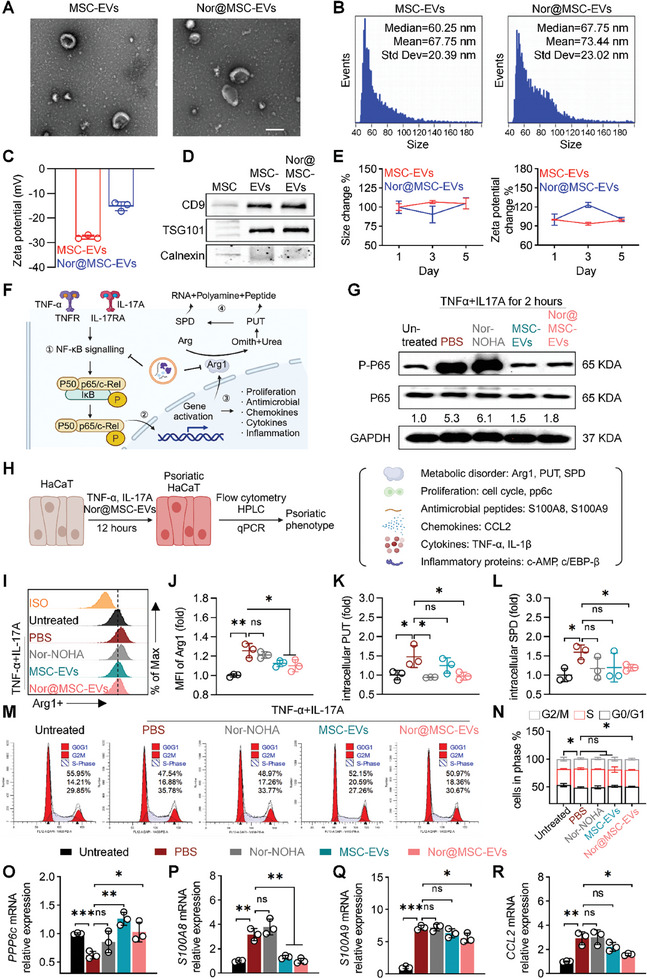
Engineering and characterizations of nor@MSC‐EVs and its modulation effect of metabolic disorder and psoriatic phenotype in keratinocytes in vitro. A) TEM images of MSC‐EVs and nor@MSC‐EVs (scale bar = 200 nm). B) Size of MSC‐EVs and nor@MSC‐EVs measured by nanoflow. C) Zeta potentials of MSC‐EVs and nor@MSC‐EVs measured by DLS. D) Expression of the marker proteins in the MSC, MSC‐EVs, and nor@MSC‐EVs analyzed by western blotting. E) size and zeta potential changes of MSC‐EVs and nor@MSC‐EVs in PBS, 4 °C analyzed via DLS. F) Schematic diagram of the NF‐κB signaling pathway activated by TNF‐α and IL‐17A in HaCaT and the inhibitory effect of nor@MSC‐EVs. G) Western blotting analysis of p‐p65 and p65 (the ratio of p‐p65/p65 indicated the activation degree of the NF‐κB signaling pathway) in HaCaT treated with different groups for 12 h, following TNF‐α and IL‐17A stimulation for another 2 h. H) Schematic diagram illustrating the induction of the HaCaT psoriatic phenotype. I) and J) Flow cytometry analysis depicting Arg1 expression in psoriatic HaCaT following treatment with nor@MSC‐EVs (n = 3). K) and L) HPLC analysis of the concentration of intracellular PUT (K) and SPD (L) in psoriatic HaCaT treated with nor@MSC‐EVs for 12 h (n = 3). M) and N) Flow cytometry analysis illustrating the cell cycle of psoriatic HaCaT treated with nor@MSC‐EVs (n = 3). O‐R) qPCR evaluated the effect of nor@MSC‐EVs on the psoriatic HaCaT, including proliferation (O), antimicrobial peptides (P and Q), and chemokines (R). All data are expressed as mean ± S.D. (n = 3). Statistical significance was calculated via one‐way ANOVA with a Tukey's test. ns, not significant; *p < 0.05, **p < 0.01, ***p < 0.001.

Subsequently, MSC‐EVs were isolated and loaded with nor‐NOHA via electroporation to obtain the engineered MSC‐EVs (nor@MSC‐EVs) (Figure S3, Supporting Information). Both the MSC‐EVs and nor@MSC‐EVs showed the double concave discs as typical shapes of EVs under transmission electron microscope (TEM) (**Figure** **3**A). Nanoflow analysis revealed a peak diameter of 60.2 nm for MSC‐EVs, and 67.7 nm for nor@MSC‐EVs (Figure 3B). Notably, the incorporation of nor‐NOHA, a positively charged small molecule, resulted in a significant zeta potential change, shifting from ‐27.8 mV of MSC‐EVs to ‐15.2 mV of nor@MSC‐EVs (Figure 3C), confirming the successful loading of nor‐NOHA into MSC‐EVs. We then quantified the unloaded nor‐NOHA via a ninhydrin reaction and calculated the encapsulation efficiency and drug loading efficiency, which were 52% and 34%, respectively (Table S1, Supporting Information). Moreover, western blotting analysis revealed that both MSC‐EVs and nor@MSC‐EVs expressed the common EV biomarkers, such as CD9 and TSG101, while showing negative expression of calnexin (Figure 3D), further validating the successful purification and modification of MSC‐EVs. Stability assessments of MSC‐EVs and nor@MSC‐EVs in PBS at 4 °C in a 5‐day period showed no significant alteration in physicochemical properties (Figure 3E). Briefly, our results convincingly demonstrated the successful isolation of MSC‐EVs and the subsequent engineering of nor@MSC‐EVs.

### 2.4 Nor@MSC‐EVs Alleviated NF‐κB Signaling Pathway‐Mediated Arg1/Polyamine Metabolic Disorder and Psoriatic Phenotype of Keratinocytes In Vitro

The combination of TNF‐α and IL‐17A has a pivotal role in the inflammatory cascade of psoriatic keratinocytes,^[^ ^19^ ^]^ where they activate the NF‐κB signaling pathway, leading to the overexpression of downstream genes, including *Arg1* and psoriasis‐related genes. It results in dysregulation of polyamine metabolism and manifests the psoriatic phenotype of keratinocytes^[^ ^13^, ^20^ ^]^ (Figure 3F). Therefore, this cytokine combination has often been employed to establish an in vitro model of psoriatic keratinocytes.^[^ ^21^ ^]^ HaCaTs were first incubated with TNF‐α and IL‐17A to trigger NF‐κB activation, then they were treated with PBS, nor‐NOHA, MSC‐EVs, and nor@MSC‐EVs. Western blotting analysis demonstrated a noteworthy increase in the p‐p65/p65 of untreated group compared to PBS group. Also, significant reduction in the p‐p65/p65 in both the MSC‐EVs group and the nor@MSC‐EVs group was observed compared to the PBS group and nor‐NOHA group, underlying the inhibitory effect of both MSC‐EVs and nor@MSC‐EVs on NF‐κB activation (Figure 3G). Further, this attenuation of NF‐κB might contribute to the decrease in Arg1‐mediated polyamine production and expression of genes associated with psoriatic phenotype (Figure 3F).

To validate the influence of nor@MSC‐EVs on Arg1‐mediated polyamine synthesis and psoriasis‐related gene expression, psoriatic HaCaT cells were incubated with different treatments before comprehensive analysis (Figure 3H). Flow cytometry results demonstrated both MSC‐EVs and nor@MSC‐EVs effectively suppressed the expression of Arg1 compared to other groups (Figure 3I,J). No evident difference was observed between the PBS group and the nor‐NOHA group, suggesting that nor‐NOHA alone cannot inhibit Arg1 overexpression. HPLC analysis further validated the decrease of intracellular polyamine levels in the nor‐NOHA group and MSC‐EVs group (Figure 3K,L). Notably, both PUT and SPD were clearly reduced in the nor@MSC‐EVs group, while it was not the same case for nor‐NOHA and MSC‐EVs groups, suggesting that nor@MSC‐EVs exhibited the most potent inhibition of both the function and expression of Arg1 as illustrated in Figure 3F.

Keratinocytes, as the targets of inflammatory cytokine cascade triggered by the over‐activated immune system, were marked by aberrant proliferation and significantly elevated levels of antimicrobial peptides, chemokines, cytokines, and inflammatory proteins^[^ ^22^ ^]^ (Figure 3H). To test the impact of nor@MSC‐EVs on the hyperproliferation of keratinocytes, a cell cycle analysis of the S phase was conducted.^[^ ^20^ ^]^ Our results showed an evident reduction in the percentage of cells in the S phase, from 35.8% in the PBS group to 30.7% in the nor@MSC‐EVs group, indicating that nor@MSC‐EVs effectively restricted the progression from G1 to S phase (Figure 3M,N). Additionally, qPCR results demonstrated an upregulation of the *PPP6C* gene, known to negatively regulate the G1‐S phase transition, increasing from 0.6‐fold in the PBS group to 1.0‐fold in the nor@MSC‐EVs group (Figure 3O), validating the observed decrease in S phase percentage. Together, these findings indicated that nor@MSC‐EVs inhibited keratinocyte hyperproliferation, offering potential therapeutic implications for psoriasis. Antimicrobial peptides produced by keratinocytes, such as S100A8 and S100A9, modulated psoriatic skin inflammation by regulating the expression of complement factor C3.^[^ ^23^ ^]^ A detailed analysis using qPCR revealed a robust decrease in the expression of both *S100A8* and *S100A9* in the nor@MSC‐EVs group (Figure 3P,Q). Psoriatic keratinocytes can secrete chemokines like CCL2 to recruit innate immune cells, including CCR2+ DCs and macrophages to the dermis.^[^ ^24^ ^]^ In this study, *CCL2* expression decreased to almost 50% in the nor@MSC‐EVs group than the PBS group (Figure 3R). Additionally, inflammatory cytokines, such as TNF‐α and IL‐1β, secreted by psoriatic keratinocytes were known to exacerbate skin inflammation.^[^ ^22^ ^]^ Our results demonstrated a significant downregulation of *TNF* and *IL1B* genes in the nor@MSC‐EVs group than the PBS group (Figure S4A,B, Supporting Information). We also assessed the expression of inflammatory proteins CAMP and CEBP/β, which contributed to the pathogenesis of psoriasis by amplifying inflammatory signaling pathways in keratinocytes.^[^ ^13^, ^21^ ^]^ The gene expression of both *CAMP* and *CEBPB* markedly decreased to 0.7‐fold of *CAMP* (Figure S4C, Supporting Information) and 1.2‐fold of *CEBPB* (Figure S4D, Supporting Information) in the nor@MSC‐EVs group compared to 1.6‐fold and 1.4‐fold in the PBS group. Collectively, these findings provided compelling evidence that nor@MSC‐EVs mitigated the psoriatic phenotype by inhibiting the expression of associated antimicrobial peptides, chemokines, cytokines, and inflammatory proteins, as well as those related to polyamine production and cell proliferation.

### 2.5 Nor@MSC‐EVs Directly Modulated Immunological Disorder In Vitro

**Figure 4:**
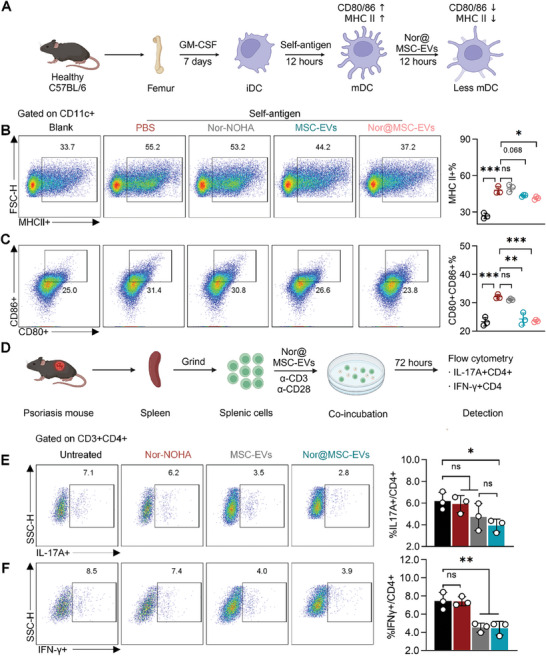
Nor@MSC‐EVs directly modulated immunological disorder in vitro. A) Schematic diagram of isolation, culture, stimulation, and treatment of BMDCs. B) Flow cytometry analysis of the expression MHC II+ in BMDCs treated with different groups. C) Flow cytometry analysis of the expression of CD80+CD86+ in BMDCs treated by different groups. D) Schematic diagram of the isolation, culture, treatment of splenic cells from psoriasis mice. E) and F) Flow cytometry analysis of the percentage of IL‐17A+CD4+ cells (E) and IFN‐γ+ cells (F) treated with different groups. All data are expressed as mean ± S.D. (n = 3). Statistical significance was calculated via one‐way ANOVA with a Tukey's test. ns, not significant; *p < 0.05, **p < 0.01, ***p < 0.001.

In addition to modulating polyamine metabolic disorder by scavenging self‐antigen to reduce BMDCs maturation, previous studies have also reported that MSC‐EVs can directly suppress BMDCs maturation.^[^ ^25^ ^]^ To this, BMDCs were stimulated with RNA+polyamine+peptide complex, then incubated with PBS, nor‐NOHA, MSC‐EVs, or nor@MSC‐EVs (**Figure** **4**A). Maturation markers of BMDCs were assessed via flow cytometry (gating strategy was shown in Figure S5, Supporting Information). Upon stimulation, a robust increase of MHC II was found in PBS group compared to the blank control, suggesting the successful induction of BMDCs maturation. The expression of MHC II decreased from 55.2% in PBS group to 44.2% in MSC‐EVs and 37.2% in nor@MSC‐EVs (Figure 4B). Additionally, co‐stimulatory molecules CD80 and CD86 were also significantly reduced after the treatment of MSC‐EVs and nor@MSC‐EVs compared to the PBS group (Figure 4C; Figure S6, Supporting Information). Nor‐NOHA alone had negligible impact on BMDCs maturation compared to the PBS group.

The pathogenesis of psoriasis is distinctively characterized by the aberrant activation of the adaptive immune system, including Th1 and Th17 cells, culminating in an inflammatory cascade that leads to hyperproliferation and erythema of the epidermis.^[^ ^3^ ^]^ To this, splenic cells isolated from IMQ‐induced psoriasis mice were incubated with nor‐NOHA, MSC‐EVs, or nor@MSC‐EVs in the presence of anti‐CD3/28 antibody (Figure 4D). It revealed that nor@MSC‐EVs significantly decreased the percentage of IL‐17A+CD4+ T cells (Th17) from 7.1% to 2.8% compared to the untreated control (Figure 4E, gating strategy was shown in Figure S7, Supporting Information). Besides, the percentage of IFNγ+CD4+ T cells (Th1 cells) notably reduced in both MSC‐EVs and nor@MSC‐EVs groups, from 8.5% to 4.0% and 3.9% respectively (Figure 4F). Conversely, nor‐NOHA alone had negligible effects on the differentiation of these T help cells. Overall, these findings supported that MSC‐EVs and nor@MSC‐EVs can directly inhibit the maturation of BMDCs and the differentiation of Th1 and Th17 in vitro, exerting direct immunomodulatory effects. The engineering process of MSC‐EVs didn't alter their immunomodulatory capability.

### 2.6 In Vivo Bioidistribution and Therapy Study of nor@MSC‐EVs in IMQ‐Induced Psoriasis Mice

To evaluate the distribution of nor@MSC‐EVs, we first use DiR dye to label nor@MSC‐EVs. In vivo imaging system (IVIS) was then applied to visualize the distribution of DiR‐nor@MSC‐EVs in both normal and IMQ‐induced psoriasis mice. Consistent with previous reports,^[^ ^9^ ^]^ we observed that DiR‐nor@MSC‐EVs accumulated in the lesional skins of psoriasis mice and rarely appeared in healthy skins in normal mice (Figure S8, Supporting Information). Compared with free DiR, the DiR‐nor@MSC‐EVs showed 2.3‐fold accumulation in the lesional skin of psoriasis mice (Figure S9B,C, Supporting Information), indicating the targeted accumulation of nor@MSC‐EVs at the inflammation site. To further investigate whether nor@MSC‐EVs could be taken up by keratinocytes in psoriasis mice, we collected the lesional skin and performed immunofluorescence analysis 48 h after tail vein injection of free Dil and Dil‐nor@MSC‐EVs (Figure S9A, Supporting Information). The Dil‐nor@MSC‐EVs (pink) were found in the epidermis, especially in pan‐keratin‐positive keratinocytes (green) (Figure S9D,E, Supporting Information).

**Figure 5:**
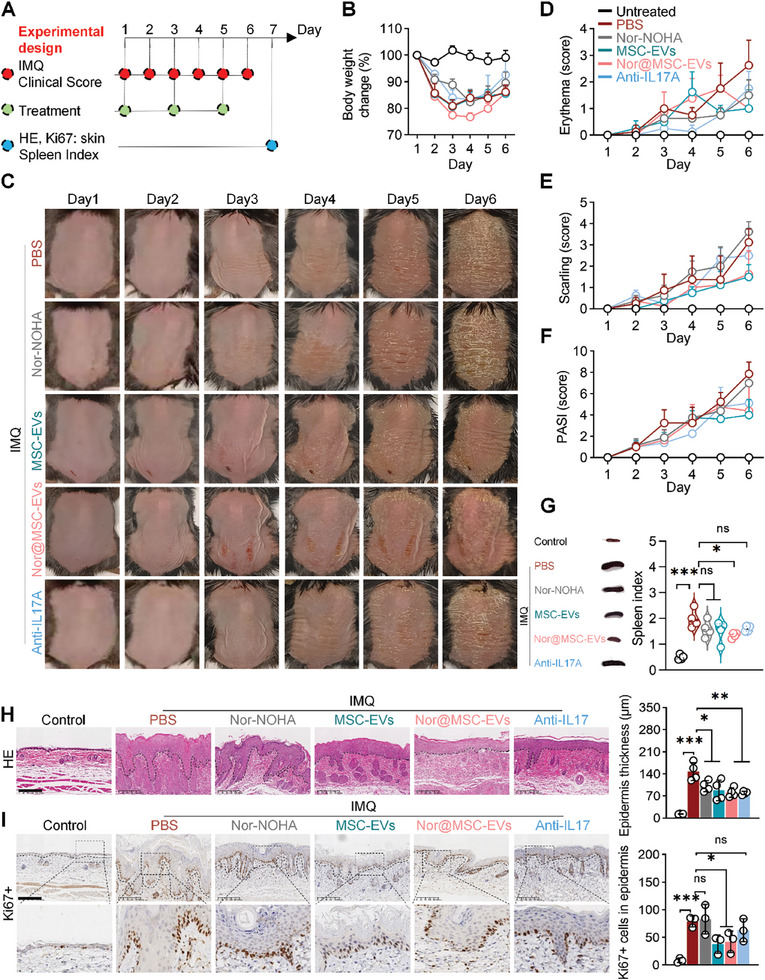
In vivo therapy study of nor@MSC‐EVs in IMQ‐induced psoriasis mice. A) Experimental design for therapy study in IMQ‐induced psoriasis mouse model. B) Body weight change (%) during the treatment (n = 4). C) Representative images of mice dorsal skins from day 1 to 6. D‐F) Corresponding clinical scores including erythema (D), scaling (E), and total (PASI) (F) measured from days 1 to 6 (n = 4). G) Spleen index of different groups after treatment (n = 4). H) Analysis of epidermis thickness in H&E‐stained sections. Scale bar = 200 µm (n = 4). I) Immunostaining of Ki67 in skin lesions and quantification of Ki67+ cells in epidermis. Scale bar = 200 µm (n = 3). All data are expressed as mean ± S.D. (n≥3). Statistical significance was calculated via one‐way ANOVA with a Tukey's test. ns, not significant; *p < 0.05, **p < 0.01, ***p < 0.001.

To assess the in vivo therapeutic efficacy of nor@MSC‐EVs, IMQ‐induced mice were administered with PBS, nor‐NOHA, MSC‐EVs, nor@MSC‐EVs, or anti‐IL17A (a clinically used bioagent for psoriasis therapy) intravenously on day 1, 3, 5^[^ ^26^ ^]^ (**Figure** **5**A). The biosafety was first assessed. Histological analysis of heart, liver, spleen, lung, and kidney tissues stained with H&E showed no signs of tissue or organ damage in all groups (Figure S10A, Supporting Information). To further evaluate hepatotoxicity and nephrotoxicity, we assessed serum levels of alanine transaminase (ALT), aspartate transaminase (AST), urea, and creatinine (CRE) (Figure S10B–E, Supporting Information). These markers in the nor@MSC‐EVs groups were close to those in the untreated group, indicating excellent biosafety and biocompatibility of nor@MSC‐EVs in psoriasis mice. The mouse body weight change, clinical score, spleen index, H&E‐staining, and Ki67‐staining were evaluated pre‐and post‐treatment (Figure 5A).

No evident body weight changes were observed among the PBS, nor‐NOHA, MSC‐EVs, nor@MSC‐EVs, and anti‐IL17A groups after topical application of IMQ cream for 6 days (Figure 5B). The MSC‐EVs, nor@MSC‐EVs, and anti‐IL17A groups displayed notable improvements in skin lesions compared to the PBS and nor‐NOHA groups (Figure 5C), characterized by reduced redness (Figure 5D) and scaling (Figure 5E) as well as lower PASI scores (Figure 5F),^[^ ^27^ ^]^ suggesting a potential alleviation in the hyperproliferation and inflammation of keratinocytes. Spleen enlargement, indicating immune cell clonal expansion, served as a sign of systemic immune response.^[^ ^26^ ^]^ Although the spleen index (spleen weight/body weight) trended downward in the nor‐NOHA, MSC‐EVs, nor@MSC‐EVs, and anti‐IL17A groups, only the nor@MSC‐EVs group exhibited a statistically significant reduction (P < 0.05) compared to the PBS group (Figure 5G), highlighting the effective inhibition of systemic inflammation by nor@MSC‐EVs.

Histological analysis revealed a marked increase in average epidermis thickness of 147.2 µm in the PBS group than 12.9 µm in the control group (Figure 5H). Post‐treatment, all groups showed significant reduction in epidermis thickness, with nor@MSC‐EVs group (81.0 µm) and anti‐IL17A group (79.6 µm) displaying the most potent suppression of epidermis hyperplasia. Furthermore, quantification of Ki67+ cells in the epidermis, a marker of cell proliferation, revealed a substantial decrease in the MSC‐EVs and nor@MSC‐EVs groups compared to the PBS group, while no statistical difference was found among other groups (Figure 5I), suggesting that the hyperproliferation of basal keratinocytes was significantly attenuated in the presence of MSC‐EVs or nor@MSC‐EVs. Collectively, a comprehensive evaluation including clinical scores, spleen index, and histological analysis indicated that nor@MSC‐EVs were the most efficacious therapeutic option for mitigating psoriasis symptoms in the IMQ‐induced mouse model.

### 2.7 Nor@MSC‐EVs Suppressed NF‐κB and Cytokines Signaling Pathway by Bulk RNA‐Sequencing Analysis

**Figure 6:**
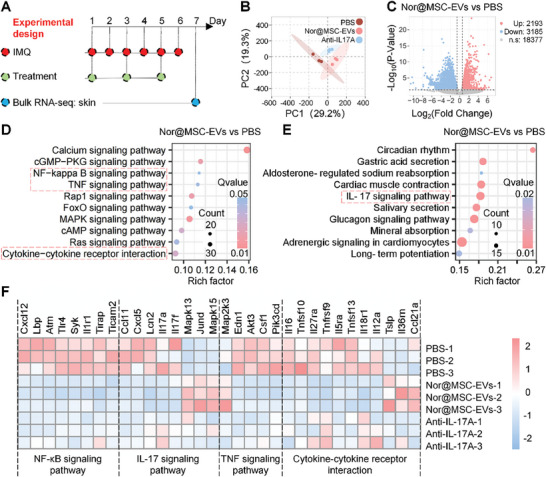
Treatment with nor@MSC‐EVs resulted in transcriptomic alterations and suppression of NF‐κB and cytokines signaling pathway in psoriasis mice skin. A) Experimental schedule for analyzing the transcriptome of psoriasis mouse skin. B) PCA of genes in PBS, nor@MSC‐EVs, and anti‐IL17A groups, n = 3. C) Volcano plot of DEGs in nor@MSC‐EVs and PBS groups (P<0.05 and |log2(fold change) |>1). D) and E) KEGG pathway enrichment analysis by DEGs in environmental information processing (D) and organismal systems (E), orb size indicates the number of genes enriched in the pathway. F) Heatmap of DEGs involved in NF‐κB signaling pathway, TNF‐α signaling pathway, IL‐17 signaling pathway, and cytokine‐cytokine receptor interactions.

To elucidate the mechanisms underlying the therapeutic effects of nor@MSC‐EVs in a psoriasis mouse model, bulk RNA sequencing was conducted on skin lesions from psoriasis mice treated with PBS, nor@MSC‐EVs, and anti‐IL17A (**Figure** **6**A). Principal components analysis (PCA) revealed distinct gene expression patterns among the groups (Figure 6B). In total, we identified 5378 significantly differentially expressed genes (DEGs), including 2193 upregulated and 3185 downregulated genes in the nor@MSC‐EVs group compared to the PBS group (Figure 6C). Among the top 10 downregulated and top 10 upregulated DEG (Figure S11, Supporting Information), we identified several key genes related to psoriasis, including *Krt25, Krt8, Muc16, Slc5a7*, *Reg3b*. *Krt25* and *Krt8*, which are critical structural proteins in the epidermis, essential for the proliferation and keratosis of keratinocytes.^[^ ^28^ ^]^ Their downregulation indicated that nor@MSC‐EVs treatment normalized epidermal thickening and keratosis, aligning with histological observations (Figure 5H,I). Epidermis barrier depends on the proper progression of the keratosis of keratinocytes. Further analysis of epidermis barrier function revealed that *Muc16*,^[^ ^29^ ^]^ which encodes an epithelial protein marked as compromised barrier function in psoriasis, was significantly downregulated following nor@MSC‐EVs treatment compared to PBS. Patients suffering from guttate psoriasis have shown positive responses to anti‐cholinergic treatment, which inhibits mast cell degranulation triggered by acetylcholine (ACh). *Slc5a7*, involved in the synthesis and transportation of ACh,^[^ ^30^ ^]^ was downregulated after nor@MSC‐EVs treatment, suggesting nor@MSC‐EVs might inhibit mast cell degranulation by reducing Slc5a7‐mediated ACh synthesis. Moreover, *Reg3b*, which promotes and sustains STAT3 activation in a self‐amplifying feed‐forward loop,^[^ ^31^ ^]^ was downregulated following nor@MSC‐EVs treatment compared to the control. It suggested that nor@MSC‐EVs may inhibit STAT3‐mediated Th17 activation,^[^ ^32^ ^]^ offering a reasonable explanation for the observed reduction in splenomegaly of psoriasis mice (Figure 5G) and further hint at investigating the immunological modulation mechanism of nor@MSC‐EVs treatment for psoriasis.

To gain deeper insights, we subjected the DEGs between the nor@MSC‐EVs and PBS groups to Kyoto Encyclopedia of Genes and Genomes (KEGG) annotation analysis. This revealed an enrichment of the top 10 signaling pathways in environmental information processing (Figure 6D) and organismal systems (Figure 6E). It showed that the significant enrichment of DEGs in pathways was associated with the NF‐κB, TNF‐α, IL‐17 signaling pathways and cytokine‐cytokine receptor interactions (Figure 6D,E). Upstream genes of the NF‐κB signaling pathway, including *Cxcl12, Lbp, Atm, Tlr4, Syk, Llr1, Tlrap, and Ticam2*,^[^ ^33^ ^]^ as illustrated in Figure 6F, were markedly downregulated following treatment with nor@MSC‐EVs and anti‐IL‐17A. This indicated a reduced activation of the NF‐κB signaling pathway which further inhibited the synthesis of Arg1 and the psoriatic phenotype of keratinocytes, as shown in Figure 3F. Additionally, both TNF‐α and IL‐17 signaling pathways are crucial in the inflammatory processes of psoriasis, leading to hyperinflammation in keratinocytes and attracting additional immune cells to the skin. The heatmap demonstrated a predominant downregulation of genes associated with these pathways in response to nor@MSC‐EVs and anti‐IL‐17A treatment (Figure 6F), suggesting a suppressive effect on TNF‐α and IL‐17A signaling and cytokine processes. These findings offer insights into the therapeutic mechanisms of nor@MSC‐EVs, partially explaining their efficacy in reducing erythema and scaling of psoriatic skin (Figure 5C–F) and the infiltration of immune cells observed in H&E staining from psoriasis mice (Figure 5H). Subsequently, we verified the expression of DEGs in NF‐κB (Figure S12, Supporting Information), IL‐17 (Figure S13, Supporting Information), and TNF (Figure S14, Supporting Information) signaling pathways, as well as cytokine‐cytokine receptor interaction (Figure S15, Supporting Information) by RT‐QPCR. The expression trends of most differentially expressed genes were consistent with the bulk RNA‐seq, although some genes didn't show statistic difference.

### 2.8 Decreased Polyamine Levels in Epidermis and Plasma Following nor@MSC‐EVs Treatment

**Figure 7:**
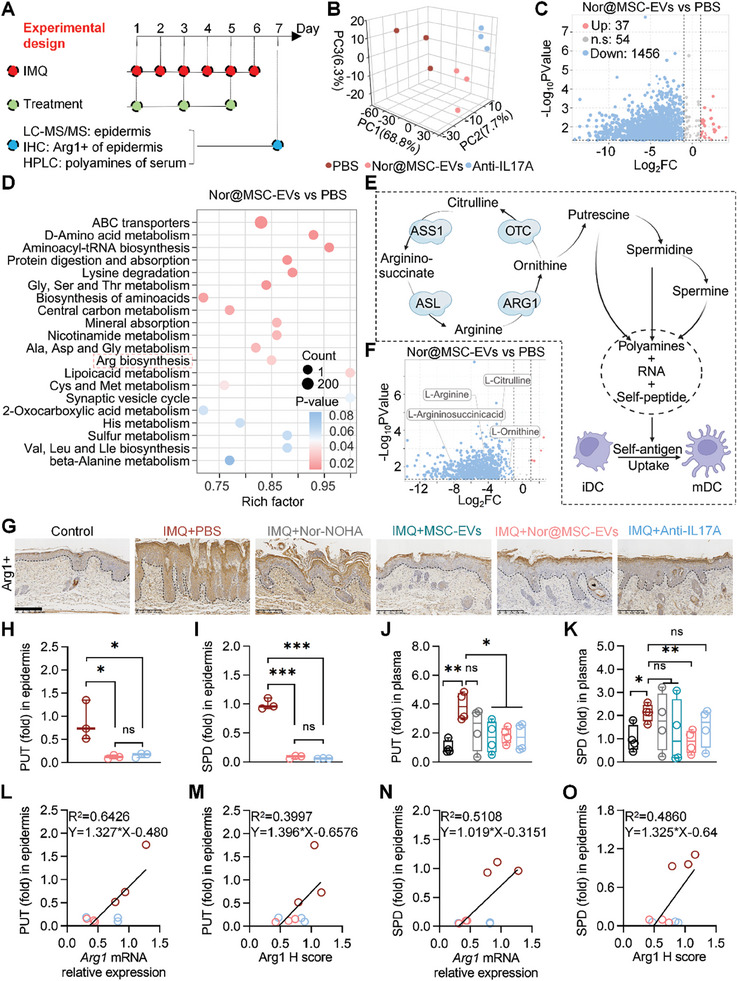
Decreased polyamine levels in epidermis and plasma following nor@MSC‐EVs treatment. A) Experimental schedule for analyzing local and systemic metabolites in the epidermis and plasma of IMQ‐induced psoriasis mice. B) PCA illustrating global sample distribution profiles and relationships among the PBS, nor@MSC‐EVs, and anti‐IL17A groups. n = 3. C) Volcano plot representing differentially regulated metabolites (DRMs) for the nor@MSC‐EVs group compared to the PBS group, with statistical significance defined as P < 0.05, |Log2(fold change)| > 2, and VIP > 1. D) Top 20 enriched metabolic pathways identified through KEGG annotation of DRMs between the nor@MSC‐EVs and PBS groups, the number of differential metabolites contained in each pathway was indicated by the size of the circle, and significance was indicated by the different color. E) Diagram illustrating arginine biosynthesis, downstream polyamine metabolism, and the influence on immune homeostasis. F) Volcano plot of DRMs of amino acids and their metabolites for nor@MSC‐EVs compared to PBS, with statistical significance defined as P < 0.05, |Log2(fold change)| > 2, and VIP > 1. G) IHC of Arg1 in the epidermis from IMQ‐induced psoriasis mice. Scale bar, 200 µm. n = 3. H‐K) Results from LC‐MS/MS and HPLC showing the fold change in local (epidermis, n = 3) and systemic (plasma, n = 4) polyamines (PUT and SPD). L) and M) Linear regression analysis demonstrating the correlation between Arg1 expression levels (Arg1 mRNA relative expression and Arg1 H score) and the PUT level. N) and O) Linear regression analysis demonstrating the correlation between Arg1 expression levels (Arg1 mRNA relative expression and Arg1 H score) and the SPD level. All data are expressed as mean ± S.D. (n ≥ 3). Linear regression analysis was performed (L‐O). Statistical significance was calculated via one‐way ANOVA with a Tukey's test (H‐K). ns, not significant; *p < 0.05, **p < 0.01, ***p < 0.001.

To investigate the local and systemic metabolic alterations of psoriasis mice, we conducted a comprehensive analysis, including non‐targeted metabolomics of the epidermis, IHC of the whole skin, and HPLC analysis of the plasma samples (**Figure** **7**A).

Metabolite profiling is an effective approach to uncover genotype–phenotype interactions and their underlying mechanisms.^[^ ^34^ ^]^ PCA clearly distinguished the classifications between the PBS, nor@MSC‐EVs, and anti‐IL17A groups (Figure 7B). A subsequent volcano plot analysis revealed significantly differential regulated metabolites (DRMs) in the nor@MSC‐EVs group, with 37 metabolites upregulated and 1456 downregulated compared to the PBS group (Figure 7C), demonstrating the profound influence of nor@MSC‐EVs on the epidermis metabolites in psoriasis mice. Subsequently, KEGG enrichment analysis was employed to display the enrichment of differential metabolites in metabolic pathways (Figure 7D). Notably, DRMs were significantly enriched in the D‐amino acid metabolism, central carbon metabolism, arginine biosynthesis pathway, *etc*. A differential abundance score (DA score) analysis showed low DA scores (less than 0) among the top 20 enriched metabolic pathways, indicating downregulation of these pathways, particularly the arginine biosynthesis pathway, in the nor@MSC‐EVs group (Figure S16, Supporting Information). Integrated transcriptome and metabolome analysis revealed significant alterations in multiple biochemical pathways, particularly in arginine, proline metabolism, and arginine biosynthesis (Figure S17, Supporting Information), highlighting consistent changes in arginine metabolism, encompassing both the transcription of key metabolic pathways and their associated metabolites. To gain a deeper understanding, we mapped the arginine metabolism pathways, highlighting the key metabolites involved, particularly the substrates (argininosuccinate and arginine) and products (ornithine and citrulline) of Arg1 (Figure 7E). Volcano plot analysis confirmed the downregulation of these substrates and products in the nor@MSC‐EVs group compared to the PBS group (Figure 7F), with statistical significance defined as P<0.05, |Log2(fold change)|>2, and VIP (Variable Importance in Projection) >1. We further investigated the expression of Arg1 in the epidermis of psoriasis mice by IHC. The results showed a significant decrease in the H score of Arg1 in both the MSC‐EVs and nor@MSC‐EVs groups compared to the PBS group. Additionally, a decreasing trend was also observed in the nor‐NOHA and anti‐IL17A groups (Figure 7G; Figure S18, Supporting Information). These findings suggested that nor@MSC‐EVs effectively inhibited Arg1 expression in the epidermis of psoriasis mice, further validating their inhibitory effects observed in vitro (Figure 3I,J).

Arg1‐mediated polyamine production in keratinocytes contributed to the formation of self‐antigens, which disrupted immune homeostasis by activating the DCs (Figure 7E). To evaluate the impact of nor@MSC‐EVs on both local and systemic polyamine metabolism, we quantified PUT and SPD levels in the epidermis and the plasma. Notably, following the treatment with nor@MSC‐EVs and anti‐IL17A, a significant reduction in PUT and SPD levels was observed in the epidermis (Figure 7H,I). The application of nor@MSC‐EVs further resulted in a substantial decline in plasma PUT and SPD levels, decreasing to 1.8‐fold and 0.9‐fold, respectively, compared to 3.9‐fold and 2.1‐fold observed in the PBS group (Figure 7J,K). To confirm whether the decrease in polyamines was associated with the reduced expression of Arg1 in the epidermis under the treatment with nor@MSC‐EVs, we performed a correlation analysis integrating metabolome data (PUT and SPD levels), transcriptome data (*Arg1* mRNA expression), and IHC results (H score of Arg1). The transcriptome analysis revealed a greater reduction in *Arg1* mRNA expression following nor@MSC‐EVs treatment compared to the anti‐IL17A group and PBS group (Figure S19, Supporting Information), consistent with the H score of Arg1 in Figure S18, Supporting Information. Positive correlations were observed between polyamine levels and Arg1 expression (Figure 7L–O). It suggested concordant alterations in the transcription and translation of the key metabolic enzyme Arg1 and its associated metabolites polyamines.^[^ ^35^ ^]^ Therefore, nor@MSC‐EVs significantly decreased local and systemic polyamine levels due to their synergistic effects for Arg1, as indicated in Figure 3F.

### 2.9 Therapeutic Effect of nor@MSC‐EVs on Modulating Local and Systemic Immunological Disorder of IMQ‐Induced Psoriasis Mice

**Figure 8:**
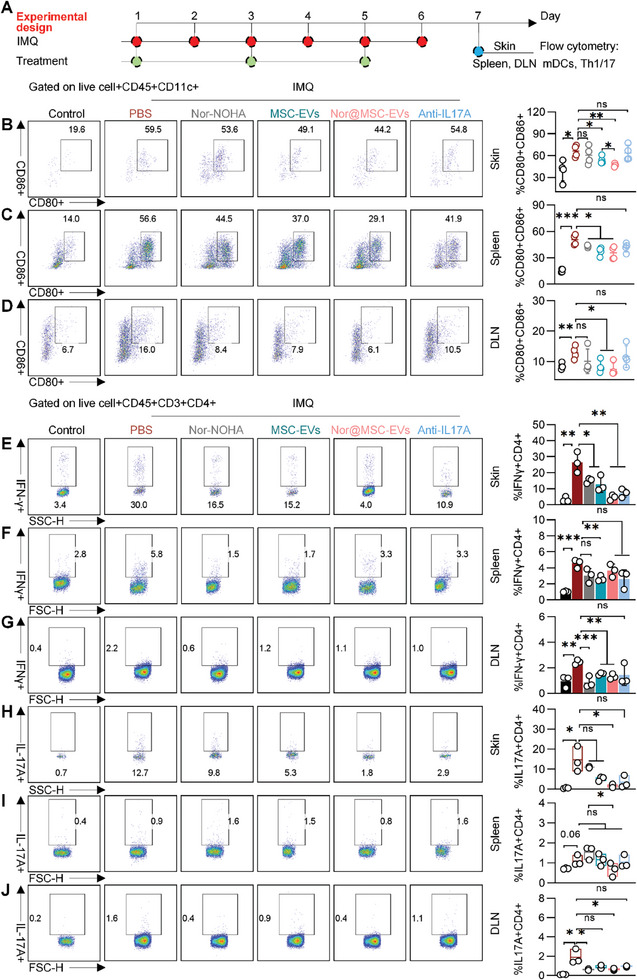
Therapeutic effect of nor@MSC‐EVs on modulating local and systemic immunological disorder of IMQ‐induced psoriasis mice. A) Experimental design for assessing the local (skin) and systemic (spleen and dLN) immunological modulation effect of PBS, nor‐NOHA, MSC‐EVs, nor@MSC‐EVs, and anti‐IL17A in IMQ‐induced psoriasis mice. Flow cytometry analysis of the percentage of CD80+CD86+ in CD45+CD11c+ cells (mDCs) in skin lesion B), spleen C), and dLN D), respectively. n = 4. Flow cytometry analysis of the percentage of IFNγ+ cells in CD45+CD3+CD4+ cells (Th1 cells) in skin lesion E), spleen F), and dLN G), respectively. n = 3. Flow cytometry analysis of the percentage of IL‐17A+ cells in CD45+CD3+CD4+ cells (Th17 cells) in skin lesion H), spleen I), and dLN J), respectively. n = 3. All data are expressed as mean ± S.D. (n≥3). Statistical significance was calculated via one‐way ANOVA with a Tukey's test. ns, not significant; *p < 0.05, **p < 0.01, ***p < 0.001.

To further explore the impact of nor@MSC‐EVs on immunological modulation in vivo, skin lesions, spleens, and draining lymph nodes (dLNs, inguinal lymph nodes) were harvested post‐treatment on day 7 to analyze the infiltrated immune cells (**Figure** **8**A).

To evaluate the local innate immune response, skin lesions were collected and processed through enzymatic digestion for flow cytometry analysis (gating strategies were shown in Figure S20, Supporting Information). A notable reduction in the proportion of CD80+CD86+ of CD11c+ cells in skin was presented, from 59.5% in the PBS group to 49.1% and 44.2% in the MSC‐EVs and nor@MSC‐EVs groups, respectively (P < 0.05) (Figure 8B). Further analysis revealed a pronounced decrease of the CD80 and CD86 expression in the nor@MSC‐EVs group, whereas the reductions observed in other groups were not statistically significant (Figure S21, Supporting Information). To assess systemic innate immune response, spleen, and dLN were collected for flow cytometry analysis (gating strategies were shown in Figure S22, Supporting Information). Compared to the PBS group, both MSC‐EVs and nor@MSC‐EVs groups displayed a reduction in the percentage of CD80+CD86+ cells of CD11c+ cells in the spleen, decreasing from 56.6% to 37.0% and 29.1%, respectively (Figure 8C). Similarly, in the dLN, this percentage declined from 16.0% to 7.9% and 6.1%, respectively (Figure 8D), indicating a significant inhibitory effect of MSC‐EVs and nor@MSC‐EVs on the maturation of DCs. Although treatment with anti‐IL17A displayed a decreasing trend in DC maturation, there was no statistical significance (Figure 8B–D).

Further investigation on the local and systemic adaptive immune response revealed a reduction of the percentage of IFNγ+CD4+ cells in skin lesion from 30.0% in the PBS group to 4.0% in the nor@MSC‐EVs group (Figure 8E). Similar phenomenon was also observed in the spleen, where IFNγ +CD4+ cells decreased from 5.8% to 3.3% (Figure 8F), and in the dLN, where they fell from 2.2% to 1.1% (Figure 8G), compared to the PBS group. Moreover, a declining trend in the percentage of IL‐17A+CD4+ cells in skin lesions was observed across the treatment groups. While the nor‐NOHA, MSC‐EVs, and anti‐IL17A groups all showed a reduction compared to the PBS group, only nor@MSC‐EVs achieved a significant decrease to 1.8% (Figure 8H). Similarly, nor@MSC‐EVs also inhibited the differentiation of IL‐17A+CD4+ cells both in spleen, from 0.9% to 0.8% (Figure 8I) and dLN, from 1.6% to 0.4% (Figure 8J) compared to the PBS group. These findings demonstrated the potent efficacy of nor@MSC‐EVs in suppressing Th1/17 cell differentiation in the skin lesion, spleen, and dLN. In summary, nor@MSC‐EVs effectively modulated both local and systemic immunological disorders in psoriasis mouse model.

## 3 Discussion

In our study, we show that Arg1 is specifically overexpressed in psoriasis by analyzing skin and plasma samples from patients with scaly erythematous dermatosis. We also demonstrate the elevation of Arg1/polyamines could lead to the overactivation of the DCs/Th17 axis triggered by excess psoriasis‐specific self‐antigens. Meanwhile, the activated Th17 cells secrete more IL‐17A and drive the overexpression of Arg1, further exacerbating the loop of Arg1/polyamine and DCs/Th17 axis (Figure 1M). To this, we introduce the engineered nor@MSC‐EVs, fabricated by incorporating Arg1 inhibitor nor‐NOHA into MSC‐EVs. We confirm first in vitro that nor@MSC‐EVs can reduce the generation of Arg1‐mediated polyamines and suppress the overactivated innate and adaptive immune responses via inhibiting DC maturation and the following Th1/17 cell differentiation. Further in vivo study with IMQ‐induced psoriasis mice indicates that nor@MSC‐EVs alleviate erythema, scaling, and thickness of skin lesions, showing a more potent therapeutic effect than the currently used clinical bioreagent anti‐IL17A. Integrating data of multi‐omics and flow cytometry show that nor@MSC‐EVs inhibit the NF‐κB signaling pathway and attenuate local and systemic metabolic and immunological disorders. Our results highlight the potential of targeting the Arg1/polyamine‐mediated DCs/Th17 axis by scavenging self‐antigens with nor@MSC‐EVs in clinically ameliorating psoriasis symptoms and providing an alternative for psoriasis therapy.

Previously, it has implied the overexpression of Arg1‐mediated the limiting of the activity of inducible nitric oxide synthase may be a molecular mechanism underlying the hyperproliferation of psoriasis.^[^ ^15^ ^]^ Lou et al. also demonstrates a rewiring of the urea‐metabolic pathway that results in increased Arg1, augmenting the release of polyamines that stabilize self‐antigen complexes, which further activate DCs.^[^ ^13^ ^]^ However, some questions remain unsolved. For instance, it is vital to prove that targeting Arg1‐mediated metabolic pathways to treat psoriasis is safe and superior to currently used clinical treatments that provide solutions not only to ameliorate but also to cure psoriasis. In consideration of the above issues to achieve synergistic therapeutic effects, we incorporate MSC‐EVs in the study design as MSC‐EVs have illustrated their great immunomodulation efficacies in treating autoimmune diseases,^[^ ^10^ ^]^ including psoriasis.^[^ ^9^ ^]^ The newest phase I/II clinical trial (ID: IRCT20080901001159N2) reveals that a dosage of 200 µg MSC‐EVs significantly improves psoriatic symptoms.^[^ ^36^ ^]^ The biological therapies targeting the IL‐23/IL‐17 axis have shown partially effective for ameliorating psoriasis symptoms, however, patients can stop responding to prolonged treatment in some cases and often find difficulties in getting fully cured, suggesting there is a need for developing alternative therapy strategies. In addition, IL‐23/IL‐17 blockade is associated with some side effects, e.g. increasing susceptibility to infections and unpredictable immune shift caused by the disrupted cytokine networks.^[^ ^37^ ^]^ Compared to the above clinically used biologic therapies, nor@MSC‐EVs could potentially avoid the overactive immune system and achieve complete healing of psoriasis by combined modulation of both metabolic and immunological disorders in psoriasis.

Careful consideration of the biosafety of nor@MSC‐EVs via intravenous administration is essential for their further translational applications. As a fact, numbers of clinical trials in humans have already evaluated the safety of intravenous MSC‐EVs administration in respiratory distress syndrome, complex regional pain syndrome, *etc*.^[^ ^41^, ^42^ ^]^ No adverse events or safety issues were reported, indicating the safety profile of MSC‐EVs. Together with our data, which assess the biosafety of intravenous administration of nor@MSC‐EVs, it provides a basis of using MSC‐EVs for psoriasis as a safe therapeutic approach.

Nevertheless, we also acknowledge that our findings are limited due to the following concerns. First, we have used only one animal model, which is not replicable to all human psoriasis features. Moreover, it is critical to thoroughly evaluate the biosafety of nor@MSC‐EVs on human. Another knotty issue is the stability of nor@MSC‐EVs for long‐term storage. Our data suggested that the physicochemical properties of nor@MSC‐EVs are not significantly altered in PBS at 4 °C for 5 days. However, the long‐term stability including the biological functions of nor@MSC‐EVs still needs to be evaluated. Collectively, future studies and clinical trials using nor@MSC‐EVs for targeting Arg1 for evaluation of both efficacies and translational possibilities for the treatment of psoriasis may be warranted.

## Conflict of Interest

The authors declare no conflict of interest.

## Author Contributions

X.Z. and B.T. contributed equally to this work. L.X., R.O., Y.X., and D.L. supervised and administrated the project. X.Z., L.X., R.O., Y.X., and D.L. conceived and designed the experiments. X.Z., B.T., Q.H., S.Y., Y.J., W.C., X.L., G.S., and Z.Y. performed the experiments. X.Z., B.T., Q.H., S.Y., Y.J., W.C., and C.Z. analyzed the data. X.Z., B.T., L.X., R.O., Y.X., and D.L. wrote the original manuscript and revised the final manuscript. All authors reviewed and given approval to the final version of the manuscript.

## Supporting information



Supporting Information

## Data Availability

The data that support the findings of this study are available from the corresponding author upon reasonable request.
