# Supplementary material for: Engineered Mesenchymal Stem Cell‐Derived Extracellular Vesicles Scavenge Self‐Antigens for Psoriasis Therapy via Modulating Metabolic and Immunological Disorders
Source: Adv Sci (Weinh). 2024 Dec 12;12(6):2410067. doi: 10.1002/advs.202410067 (PMC11809393; doi:10.1002/advs.202410067)
Supplement: Supplementary file 1 — Supporting Information [file ADVS-12-2410067-s001.docx]

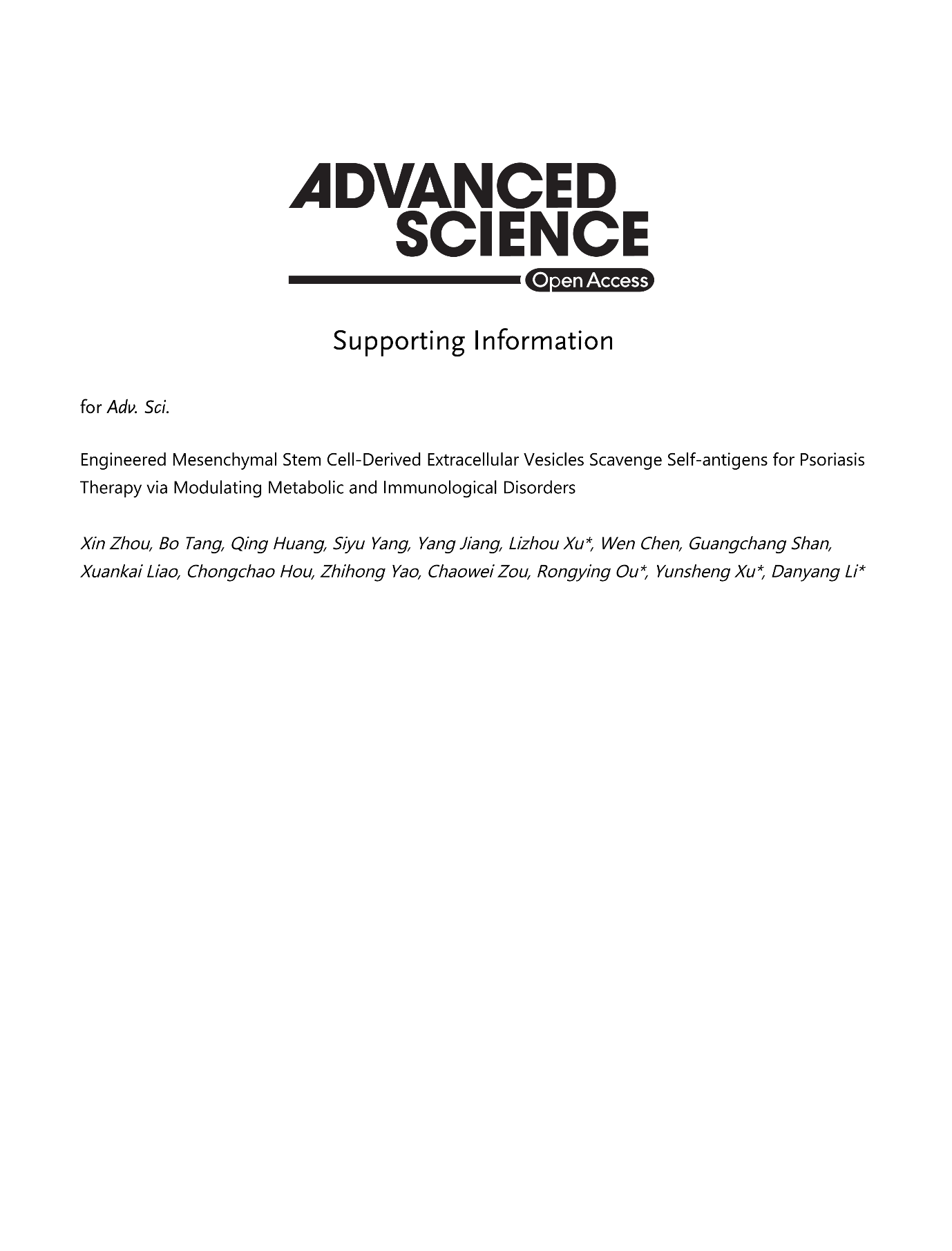


Supporting Information

**Engineered Mesenchymal Stem Cell-Derived Extracellular Vesicles Scavenge Self-antigens for Psoriasis Therapy via Modulating Metabolic and Immunological Disorders**

*Xin Zhou, Bo Tang, Qing Huang, Siyu Yang, Yang Jiang, Lizhou Xu*, Wen Chen, Guangchang Shan, Xuankai Liao, Chongchao Hou, Zhihong Yao, Chaowei Zou, Rongying Ou*, Yunsheng Xu*, Danyang Li**

**Experimental Section**

**Materials**

DMEM/low glucose medium (#C11885500BT), DMEM/high glucose medium (#C11995500BT), RPMI 1640 medium (#C11875500BT), penicillin and streptomycin (#15140122), L-glutamate (#25030-081) - purchased from GIBCO. Ficoll solution (#PB200301) - obtained from Procell. Nor-NOHA (#S9737) - supplied by Selleck. Ninhydrin hydrate (#N8650-5) - purchased from Solarbio. Sodium hydrogen carbonate (#1229GR500) - provided by Biofroxx. DiR (#D4006) - purchased from UElandy. Dil (#D775877) - purchased from Macklin. DAPI (#D9542) - purchased from Sigma-Aldrich. Antibodies including anti-human CD9 (#AF5139) - provided by Affinity Biosciences; anti-human CD63 (#25682-1-AP) and anti-human CD81 (#27855-1-AP) - provided by Proteintech; Anti-human Alix (#92880S), anti-human p-NF-κB p65 (#3033s), anti-human NF-κB p65 (#8242s), anti-human GAPDH (#2118L) - provided by CST; Anti-human TSG101 (#ab125011) - provided by Abcam; HRP-conjugated goat anti-rabbit IgG (#AS014) - provided by Abclonal; Anti-mouse Pan-cytoketatin (#53-9003-80) - provided by ThermoFisher. Anti-mouse Ki67 (#GB111499-100) and anti-mouse Arg1 (#GB11285-100) - provided by Servicebio; Anti-human Arg1 (#ZA-0615) - provided by ZSGB-Bio. Cytokines such as Human TNF-α (#300-01A-10), Human IL-17A (#200-17-25), GM-CSF (#315-03) - Obtained from PeproTech. RIPA buffer (#BL504A) and PMSF (#BL507A) - provided by Biosharp. Protease and phosphatase inhibitor cocktail (#GRF102) and 10% SDS/PAGE gel (#PG212) - supplied by EpiZyme. Trizol (#15596026) - obtained From Invitrogen. HiScipt II Kit (#R233-01) - provided by Vazyme. Cell Cycle Analysis Kit (#C1052) and red cell lysis buffer (#C3702) - obtained from Beyotime. HNRNPA1226-237 - synthesized by GenScript. MojoSort TM Mouse naïve CD4+ T Cell Isolation Kit (#480039), anti-CD28 Antibody (#102116), anti-CD3 antibody (#100340) – obtained from BioLegend. Phorbol myristate acetate (PMA) (#P8139), ionomycin (#407951), collagenase IV (#C5138-25MG) - obtained from Sigma-Aldrich. Monensin (#HY-N0150) - obtained from MCE; Dnase l (#11284932001) - obtained from Roche. For flow cytometry, Fixable Viability Stain 700 (#564997), BV510 anti-mouse-CD3 (#563024), and Cytofix/Cytoperm (#554714) - provided by BD. Anti-mouse CD16/32 (#101320), Pacific Blue anti-mouse CD45 (#103126), APC anti-mouse CD11c (#117310), FITC anti-mouse CD80 (#104705), PE/Cy7 anti-mouse CD86 (#105014), PE anti-mouse IL17A (#506903) - purchased from biolengend. BV786 anti-mouse CD4 (#417-0042-80) and PerCP/Cy5.5 anti-mouse IFN γ (#45-7311-80) - purchased from ThermoFisher.

**Ethics statement**

All analyses of human samples (skin and blood) were in full agreement with our institutional guidelines, with the approval of the Ethical Committee of the Seventh Affiliated Hospital, Sun Yat-sen University (KY-2021-039-01). All animal experiments and procedures were approved by the Institutional Animal Care and Use Committee and Laboratory Animal Welfare and Ethics Committee at Sun Yat-sen University (SYSU-IACUC-2024-000434).

**Clinical samples**

Patients enrolled in our study meet the diagnostic standards of psoriasis. The disease activity was estimated by PASI scores. Adult patients were randomly recruited from inpatient and were eligible to participate with the following rules: 1. Should not have another autoimmune or systemic disease; 2. Had not received systemic treatment in the past four weeks; 3. Had reported regular schedules and sleep-wake patterns in the past four weeks. Controls were collected from sex- and age-matched healthy volunteers.

**Culture and identification of hUC-MSCs**

Human umbilical cord-derived mesenchymal stem cells (hUC-MSCs) were donated by Wingor company (Shenzhen, Guangdong, China). Briefly, the bone marrow was diluted with DMEM/low glucose medium and was isolated with Ficoll solution after centrifugation at 800 *g* for 20 minutes. The hUC-MSCs were cultured with DMEM/low glucose containing 10% FBS that was changed every 3 days. After the cells reached 80%-90% confluence, the serum-free medium was added for 48 hours. The morphology of the cultured hUC-MSCs was observed under a microscope. The osteogenic and adipogenic differentiation potential of hUC-MSCs was identified using inductive mediums according to Alizarin Red, and Oil Red O. The typical MSC markers, including CD73 and CD105, and hematopoietic cell markers, including CD45 and CD34 were characterized with flow cytometry.

**Isolation of MSC-Evs**

The supernatant of the hUC-MSCs culture was collected and centrifuged at 1000 *g*, 2000 *g*, and 10000 *g* to remove cells, cell debris, and micro-vesicles. Furthermore, the supernatant was ultra-centrifuged at 110,000 *g* for 2 hours at 4 ℃ (Beckman Coulter, Optima XPN). The pellets were resuspended with PBS and ultra-centrifuged at 110,000 *g* for 2 hours at 4 ℃ again. Finally, the pellets were resuspended with 200 μL PBS and stored at -80 ℃.

**Preparation of nor@MSC-EVs**

Electroporation mixtures were prepared by mixing 100 μg/mL MSC-Evs and 100 μg/mL nor-NOHA in 1 mL PBS. Electroporation was performed using a Gene Pulser/MicroPulser under the following conditions: 110 V, 6 ms pulse/10 ms pause, 10 cycles, capacitance of 940 μF. Solutions were collected and incubated at 37 ℃ for 2 hours. To remove unloaded nor-NOHA, three washing steps with PBS using ultrafiltration (100 kDa MWCO, 10–20 minutes, 3000 *g*, 4℃) were performed. The purified nor@MSC-EVs was resuspended in PBS. The unloaded nor-NOHA is quantified by ninhydrin reaction for further calculating the encapsulation efficiency and drug loading efficiency using the equations below.

$$\text{Encapsulation efficiency \% = }\frac{\text{loaded drug}}{\text{total drug}}\text{ ×1}\text{00\%}$$

$$\text{Drug loading efficiency \% = }\frac{\text{loaded drug}}{\text{total drug + }\text{Evs}}\text{ ×100\%}$$

The ninhydrin reaction was performed as follows: 1. Mixing 500 μL ultrafiltrate, 120 μL 0.05 M KH2PO4 buffer (pH=6.5), and 30 μL 2% ninhydrin solution; 2. Heat the mixture in a boiling water bath for 20 minutes; 3. Quantitative analysis was performed using spectrophotometry by measuring the absorbance at 440 nm. The standard curve was presented in **Fig. S23**.

**Characterizations of nor@MSC-EVs**

Nanoparticle tracking analysis (NTA) was performed to identify the particle size distribution and concentration of Evs. Dynamic light scattering (DLS) was applied to assess the zeta potential of Evs. Western blot was employed to identify the protein markers involving CD9, CD63, CD81, Alix, and TSG101. TEM was employed to observe the morphology of Evs.

**Cells**

HaCaT and NIH/3T3 cell lines were gifts from Professor Fang Cheng (School of Pharmaceutical Sciences, Sun Yat-sen University, Shenzhen, China). HaCaT and NIH/3T3 cells were cultured in DMEM/high glucose medium complemented with 10% FBS, 1% penicillin and streptomycin. The cell culture medium was changed every two days, and cells were passaged before becoming confluent. For inducing psoriasiform keratinocytes *in vitro*, 50 ng/mL human TNF-α and 500 ng/mL human IL-17A were added into complete DMEM/high glucose medium^23^ to culture HaCaT cells for 12 hours. After inducing psoriasiform keratinocytes for 12 hours, qPCR analysis was performed to identify psoriasis related to genes as shown in **Table S2** Mouse bone marrow cells were flushed from the tibias and femurs of 8-week-old C57BL/6 mice and cultured in a T75 flask containing 15 mL RPMI 1640 medium supplemented with 10% FBS, 1% penicillin and streptomycin, 1% L-glutamate, and 20 ng/mL GM-CSF. The culture medium was changed every two days, and suspension cells were collected on day 6 for subsequent experiments.

**Western blotting**

Briefly, 400 k/well of HaCaT cells were seeded in a 6-well plate one night before the study. Then, different agents including 26 μg/mL nor-NOHA, 50 μg/mL MSC-EVs, or 50 μg/mL nor@MSC-EVs in the medium were added into the HaCaT cells for a further 12 hours of culturing. 50 ng/mL TNF-α and 500 ng/mL IL-17A were added into HaCaT cells for 2 hours before harvesting. Cells were then washed three times with PBS and lysed in RIPA buffer containing 1X protease and phosphatase inhibitor cocktail as well as 1 mM PMSF for protein extraction, subsequently the supernate was obtained after centrifuging at 12,000 *g* for 15 minutes at 4 ℃. After protein quantification by BCA assay, 10 μg/lane proteins were loaded into 10% SDS/PAGE gel. The proteins in the gel were transferred to the PVDF membrane and incubated with primary and secondary antibodies. The antibodies used in this experiment were as follows: p-NF-κB p65 (1:1000 dilution), NF-κB p65 (1:1000 dilution), GAPDH (1:1000 dilution); HRP-conjugated goat anti-rabbit IgG (1:5000 dilution). The signal was detected by ECL solution, and the image was visualized using a ChemiDoc^TM^ imaging system.

**Quantitative Real-time PCR**

Briefly, 200 k/well of HaCaT cells were seeded in 6-well plates and cultured until they reached 50% confluence. Then, different agents including 26 μg/mL nor-NOHA, 50 μg/mL MSC-EVs, or 50 μg/mL nor@MSC-EVs were added into in medium containing 50 ng/mL TNF-α and 500 ng/mL IL-17A for a further 12 hours of culturing.

Total RNA from the HaCaT cells treated with different samples described above was isolated with Trizol according to the manufacturer's instructions. RNA was reverse transcribed to cDNA using HiScipt II Kit. RT–qPCR was performed on the CFX96 system (Bio-Rad) using ChamQ Universal SYBR qPCR master mix. Gene expression levels were normalized to those of GAPDH. All primers used in this study are listed in **Table S2** and **S3**.

**Cell cycle study**

Cells were synchronized with a serum-free culture medium for 24 hours. After starvation, 60 k/well of HaCaT cells were seeded in 24-well plate 12 hours prior to the study. Subsequently, 26 μg/mL nor-NOHA, 50 μg/mL MSC-EVs, or 50 μg/mL nor@MSC-EVs were added into the medium containing 50 ng/mL TNF-α and 500 ng/mL IL-17A for another 12 hours. Following, the cells were harvested and fixed with 500 μL cold 70% ethanol (precool at -20℃). The fixed cells were incubated with propidium iodide containing RNase A according to the manufacturer's instructions of the Cell Cycle Analysis Kit. Cell cycle phases were identified and quantified using ModFitLT 5.0 Software.^43^

**Stimulation of BMDC**

Total mouse RNA (self-RNA) was extracted from the NIH/3T3 cells. A Mixture of putrescine (PUT) (Sigma-Aldrich, #P5780-5G), spermidine (SPD) (Sigma-Aldrich, # S0266-1G), and spermine (SPM) (Sigma-Aldrich, #S4264-1G) was used as polyamines. Synthetic HNRNPA_1226-237_ was used as self-peptide. To evaluate the effect of polyamines concentration on BMDCs maturation, 5 μg/mL self-RNA, 0.1, 1, or 2.5 μg/mL polyamines (PUT: SPD: SPM=1:1:1), and 1 μg/mL self-peptide were premixed as self-antigen (RNA-polyamine-peptide complex) in RPMI 1640 medium for 20 minutes at RT. To evaluate the impact of 26 μg/mL nor-NOHA, 50 μg/mL MSC-EVs or, 50 μg/mL nor@MSC-EVs on BMDCs maturation, these agents were added into RPMI 1640 medium containing the pre-mixture of 5 μg/mL self-RNA, 2.5μg/mL polyamines, and 1μg/mL self-peptide. LPS (1 μg/mL) was used as a positive control. Mouse BMDCs were seeded in 48-well plates at a density of 250 k/well. After overnight treatment, CD80, CD86, and MHC class II of CD11c+ DCs were detected by flow cytometry.^16^

**Naïve CD4+ T cell sorting and activation**

Naïve CD4+ T cells were isolated from the spleen of C57BL/6 of 6 weeks using a MojoSort TM Mouse naïve CD4+ T Cell Isolation Kit according to the manufacturer’s instruction. Meanwhile, BMDCs were treated with different combinations of self-RNA, polyamines, and self-peptide for 12 hours as described above. Naïve CD4+ T cells were co-cultured with BMDCs at 5:1 for 72 hours. Briefly, 250 k/well naïve CD4+ T cells and 50 k/well BMDCs were mixed and seeded into a 96-well plate. After 72 hours of culture, cells were stimulated with 50 ng/mL PMA and 1000 ng/mL ionomycin for 6 hours, and 5 ug/mL monensin for the last 2 hours. Finally, the percentage of IFNγ+ and IL-17A+ of CD4+CD3+ T cells was detected by flow cytometry.

**Differentiation of T cell**

For splenic cell culture with nor@MSC-EVs, the spleen from an IMQ-induced psoriasis mouse was ground on a 6 cm dish. After centrifugation (400 *g*, 6 minutes), cells were resuspended in RPMI 1640 supplemented with 10% FBS, 1% L-glutamine, 1% penicillin and streptomycin, and 2μg/mL anti-CD28 Antibody. Next, 300 k splenic cells/well were seeded into the 96-well-plate pre-coated 5 μg/mL anti-CD3 antibody. Meanwhile, 26 μg/mL nor-NOHA, 50 μg/mL MSC-EVs, and 50 μg/mL nor@MSC-EVs were added into the medium. After 72 hours of culture, cells were stimulated with 50 ng/mL PMA and 1000 ng/mL ionomycin for 6 hours, and 5 ug/mL monensin for the last 2 hours. Finally, the percentage of IFNγ+ and IL-17A+ of CD4+CD3+ T cells was detected by flow cytometry.

**Mouse model of psoriasis**

C57BL/6J mice (8 weeks old, female) were randomly assigned to a group of 5 mice and maintained in an SPF environment. For establishing mice model of psoriasis, mice received daily topical applications of 62.5 mg 5% IMQ cream on the shaved dorsal skin (2×3 cm^2^) for six consecutive days. On day 1, day 3, and day 5, all experimental groups received different therapeutic agents by intravenous injection of the tail vein, including 52 μg nor-NOHA, 100 μg MSC-EVs, and 100 μg nor@MSC-EVs and 100 μg anti-IL17A per mouse (BioXcell, #BE0173). Psoriasis mice treated with PBS were served as vehicle control. During the treatment, the weight and PASI score were monitored daily. On day 7, mice were sacrificed, and blood, skin tissue, drain lymph nodes (inguinal lymph nodes), and spleen were collected for following analysis.

**Biodistribution of nor@MSC-Evs**

To explore the biodistribution of nor@MSC-EVs, 8-week-old female C57BL/6 mice were randomly divided into two groups (Free-DiR and DiR@MSC-EVs). 200 μL of Free DiR or DiR-labelled nor@MSC-EVs (nor@MSC-EV: 100 μg, DiR: 5 μg) was intravenously injected into the mice. 48 h after injection, mice were euthanized. Using the *In Vivo* Imaging System (IVIS) with a 0.5 s exposure time (PerkinElmer, Hopkinton, MA, USA), the fluorescence intensity of heart, liver, spleen, lung, kidney and skin was assessed *ex vivo*. To explore the uptake of keratinocytes of nor@MSC-EVs, we randomly divided 8-week-old female C57BL/6 mice into two groups (Free-Dil and Dil@MSC-EVs). Immunofluorescence analysis was performed for dorsal skin section stained with anti-mouse Pan-cytokeratin and DAPI. The staining process followed the manufacturer’s instructions. Fluorescence intensity analysis was conducted by image J.

**Immunohistochemical and histological analysis**

Mice back skin was collected, fixed, embedded, sectioned (5 μm), and stained with hematoxylin and eosin (HE), anti-mouse Ki6, and anti-mouse Arg1. Clinical samples were stained with anti-human Arg1. The staining process followed the manufacturer’s instructions. Histological analysis was conducted by image J. The epidermis thickness was measured by pixel size (plotting scale as the control). Ki67+ positive cells were counted by image J. The histochemistry score (H score) was applied to evaluate the positive cases of Arg1 in mice and clinical samples, in which both the intensity and percentage of positivity were determined using the following equation: H score= 3 × (strong intensity) × % + 2 × (moderate intensity) × %+1 × (mild intensity) × %.

**High Performance Liquid Chromatography**

High-Performance Liquid Chromatography (HPLC) was utilized to determine the concentrations of polyamines in samples from HaCaT cells and plasma. Sample pre-treatment was conducted as follows: (1) HaCaT cells were washed thrice with PBS buffer; (2) plasma was separated from blood *via* centrifugation at 3000 rpm, for 15 minutes. Subsequently, samples were resuspended in 300 µL of 5% (v/v) trichloroacetic acid and heated at 100 ℃ for 15 minutes. Following heating, samples were centrifuged at 12,000 rpm for 20 minutes to obtain supernatant. 200 µL of the supernatant was transferred to a new tube containing 200 µL of 2 M NaOH and 3 µL of benzoyl chloride for derivatization. The mixture was incubated in a water bath at 37 ℃ for 25 minutes. Afterward, 300 µL of saturated NaCl solution and 350 µL of diethyl ether were added. Further centrifugation at 5,000 rpm for 15 minutes, 200 µL of the supernatant was transferred to a new tube and the diethyl ether was evaporated. The residual sample was redissolved in methanol for HPLC analysis. The HPLC system (Shimadzu, Japan) was equipped with a Nucleosil ODS column (250 × 4.6 mm, 5 µm diameter). Isocratic elution was performed using 40% solvent A (water) and 60% solvent B (methanol) at a flow rate of 1 mL/minute. A photodiode array (PDA) detector monitored the eluate at 254 nm, with the total analysis duration being 15 minutes. Concentrations of PUT and SPD were quantified based on a standard curve generated from known standards. The elution times, determined by the known standards, were identified as 4.6 minutes for PUT and 8.2 minutes for SPD.

**Transcriptome analysis**

Animal samples were extracted by Trizol method. After successful extraction, RNA was dissolved by adding 50 µL of DEPC-treated water. Subsequently, total RNA was identified and quantified using a Qubit fluorescence quantifier and a Qsep400 high throughput biofragment analyzer.

**Metabolome Analysis**

The sample stored at -80 °C refrigerator was thawed on ice. The thawed sample was homogenized by a grinder (30 HZ) for 20 s. A 400 μL solution (Methanol: Water = 7:3, V/V) containing internal standard was added in to 20 mg grinded sample, and shaked at 2500 rpm for 5 min. After placing on ice for 15 min, the sample was centrifuged at 12000 rpm for 10 min (4 °C). A 300 μL of supernatant was collected and placed in -20 °C for 30 min. The sample was then centrifuged at 12000 rpm for 3 min (4 °C). A 200 μL aliquots of supernatant were transferred for LC-MS analysis.

**Flow cytometry**

For the analysis the content of immune cells in the skin, 2×2 cm^2^ dorsal skin was cut off and washed with PBS buffer until clarification (remove IMQ cream to keep cell viability). The whole skin was cut into pieces (＜0.5 mm). Subsequently, skin pieces were digested with 2 mL basic DMEM medium containing 1 mg/mL collagenase IV and 200 μg/mL DNase l at 37 ℃ for around 60 minutes. After that, the digestion was stopped with a completed DMEM medium containing 10% FBS. Skin cell suspensions were filtered through a 70 μm cell strainer to obtain single cells. To analyze immune cells in the spleen or lymph nodes, spleen or lymph nodes were ground on the 6 cm dish containing 2 mL FACS buffer. Subsequently, the cell suspensions were filtered through a 70 μm cell strainer. The splenic single cells were lysed using red cell lysis buffer. Then, cells were stimulated with PMA and ionomycin for 6 hours and monensin for the last 2 hours.^26, 45, 46^

Cells were stained with Fixable Viability Stain 700 (1:1000, v/v) in 1 mL PBS buffer for 15 minutes at room temperature, and then incubated with anti-mouse-CD16/32 (1:50) in 50 μL FACS buffer for 10 minutes to block FcR. For surface marker staining, cells were incubated with Pacific Blue anti mouse-CD45 (1:40), BV510 anti mouse-CD3 (1:20), BV786 anti mouse-CD4 (1:40), APC anti mouse-CD11 (1:40), FITC anti mouse-CD80 (1:20), PE/Cy7 anti mouse-CD86 (1:40) in 50 μL FACS buffer for 30 minutes on ice. For intracellular cytokines staining, cells were fixed and permeabilized using BD Cytofix/Cytoperm and were incubated with PE anti mouse-IL17A (1:40) and PerCP/Cy5.5 anti mouse-IFN γ (1:40) in 50 μL FACS buffer for 45 minutes on ice. Isotype or FMO was used as the negative control. The data was acquired by Beckman cytoFlex LX and analyzed by FlowJo software 10.8.1.

**Statistical analysis**

Statistical analyses were performed using GraphPad Prism 9.5.0. Experimental data were analyzed using unpaired two-tailed t test and one-way ANOVA with a Tukey’s test. P < 0.05 (*), P < 0.01 (**), and P < 0.005 (***) were considered significant. ns indicates no significance. Error bars represent SD. Correlation analysis was made using linear regression analysis and the linear correlation index R^2^. All *in vitro* and *in vivo* experiments were repeated at least three times independently.

**Supplementary Figures**


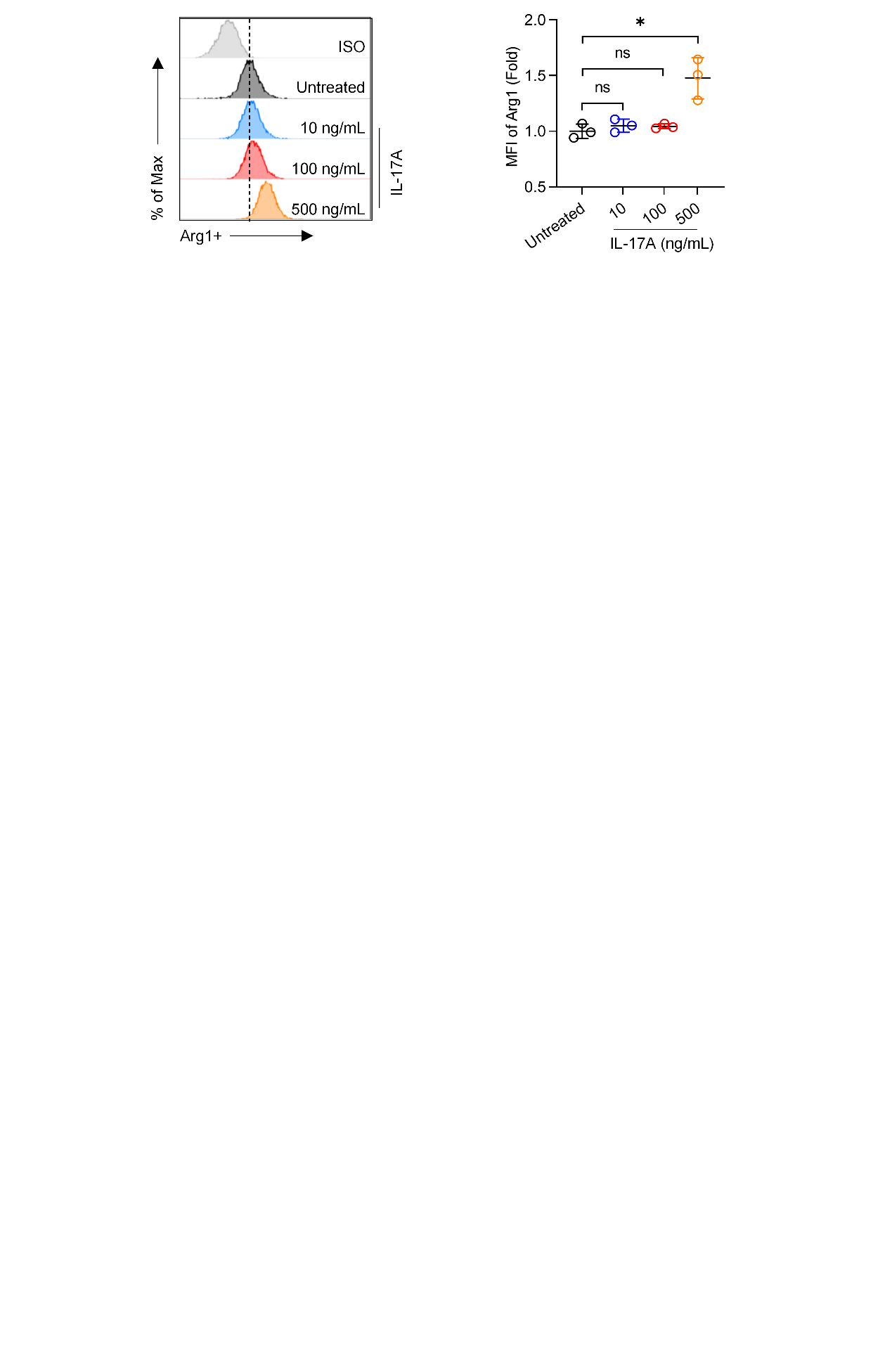


**Figure S1.** Elevated expression level of Arg1 with increasing IL-17A concentration in keratinocytes *in vitro*. Data are expressed as mean ± S.D. (n=3). Statistical significance was calculated *via* one-way ANOVA with a Tukey’s test; ns, not significant; *P<0.05.


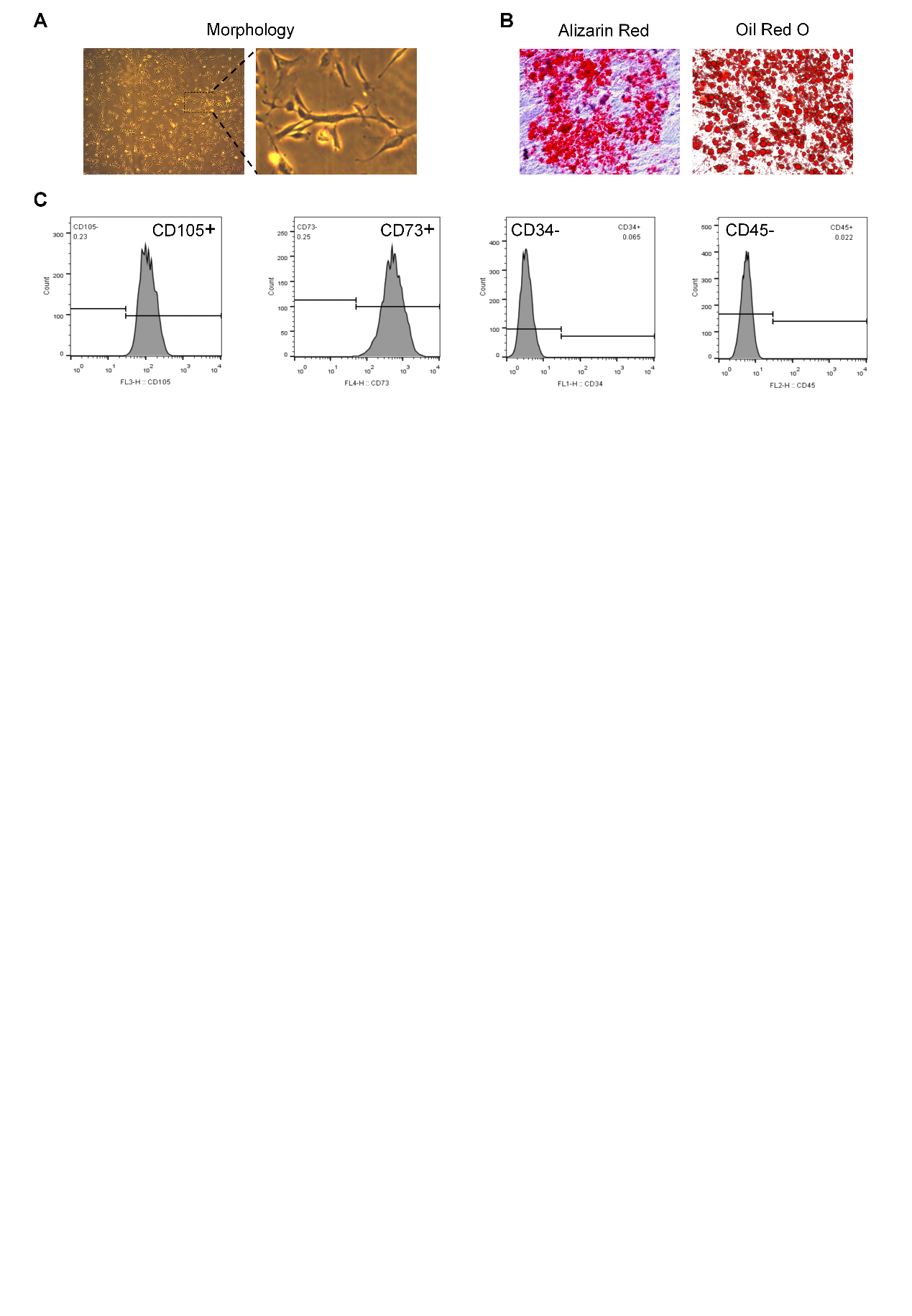


**Figure S2.** Characterizations of huc-MSC. A) The morphological identification of primary MSC after 3 days of culture. B) Alizarin Red S staining of MSC for identifying osteogenic differentiation; Oil Red O staining of MSC for identifying adipogenic differentiation. C) Flow cytometry for the positive and negative phenotypic markers of MSC.


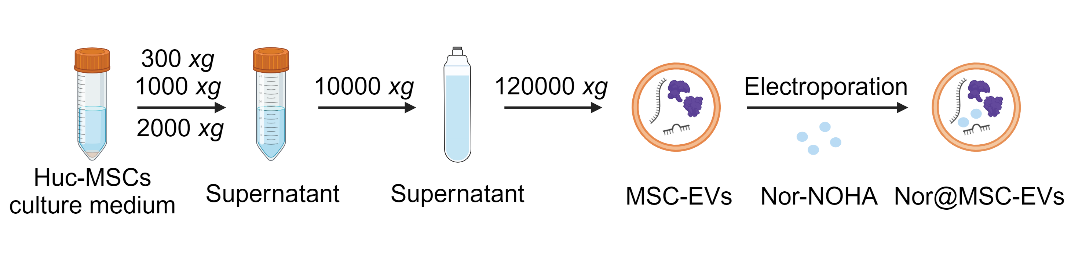


**Figure S3.** Schematic illustration showing the purification and construction procedure of nor@MSC-EVs.


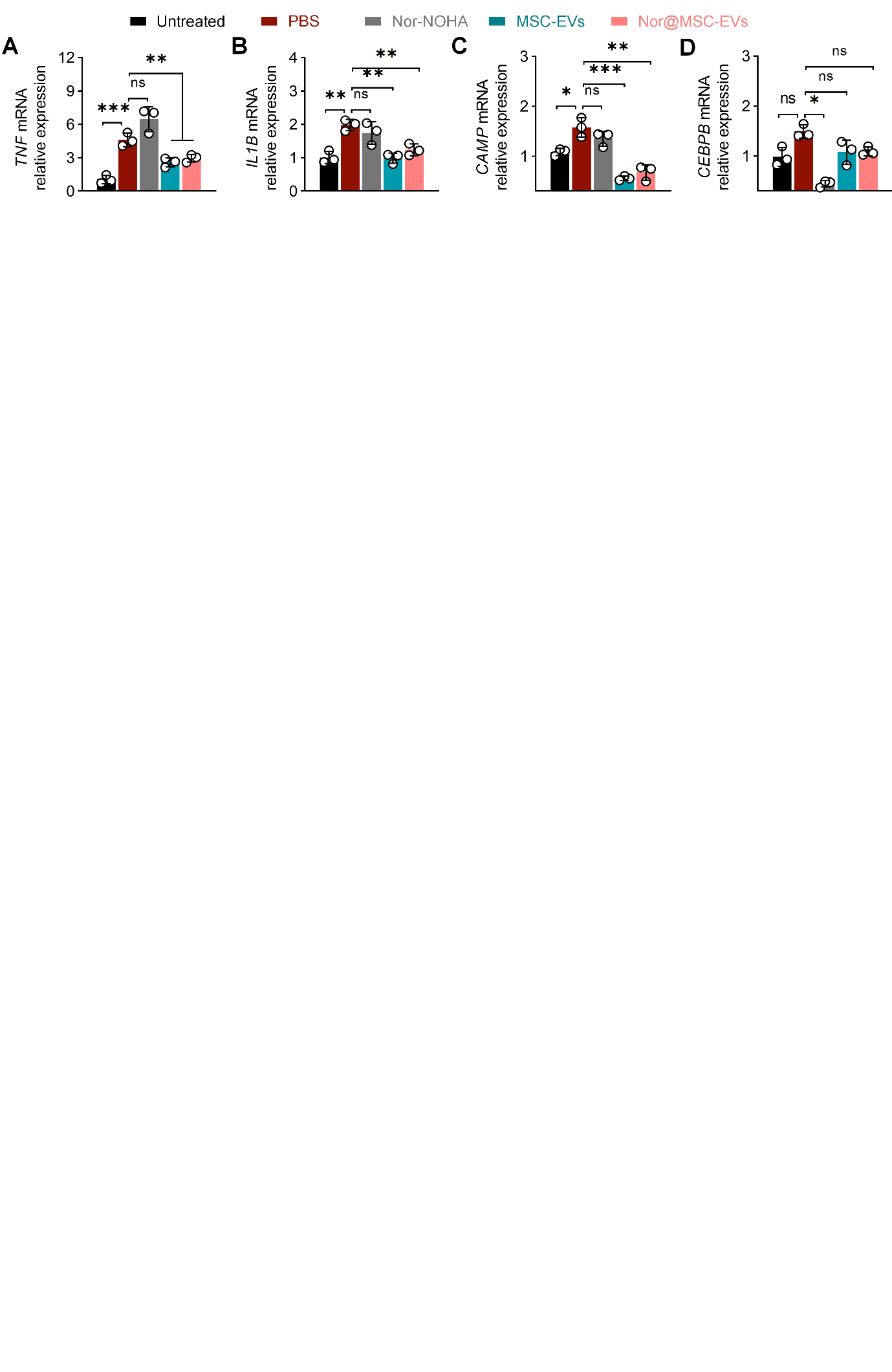


**Figure S4.** Evaluation of the effect of nor@MSC-EVs on the psoriatic phenotype of HaCaT by RT-qPCR. A) and B) cytokines; C) and D) inflammatory proteins. Data are expressed as mean ± S.D. (n=3). Statistical significance was calculated *via* one-way ANOVA with a Tukey’s test. ns, not significant; *P<0.05, **P<0.01, ***P<0.001


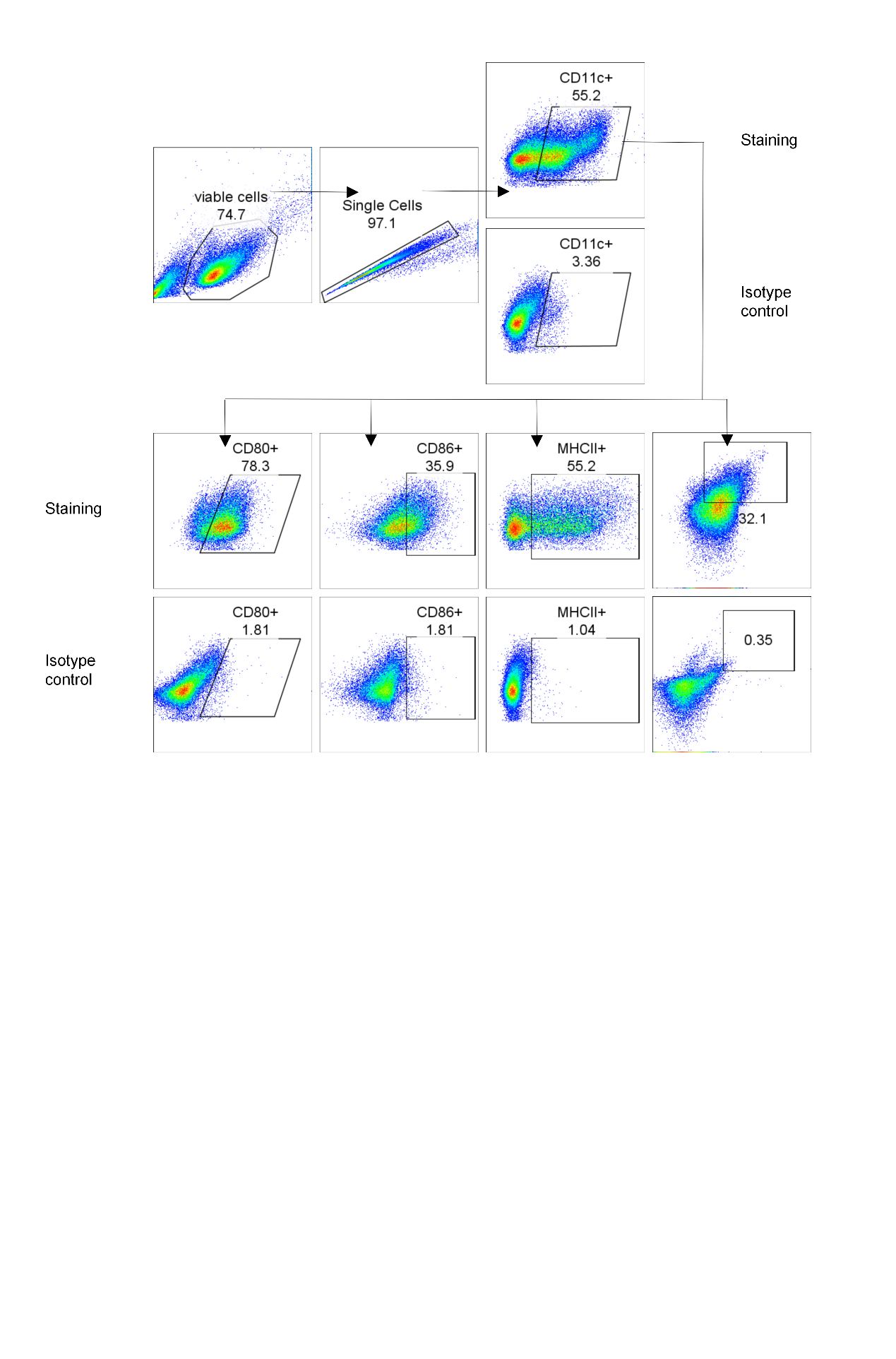


**Figure S5.** Flow cytometry gating strategy of BMDCs *in vitro.*


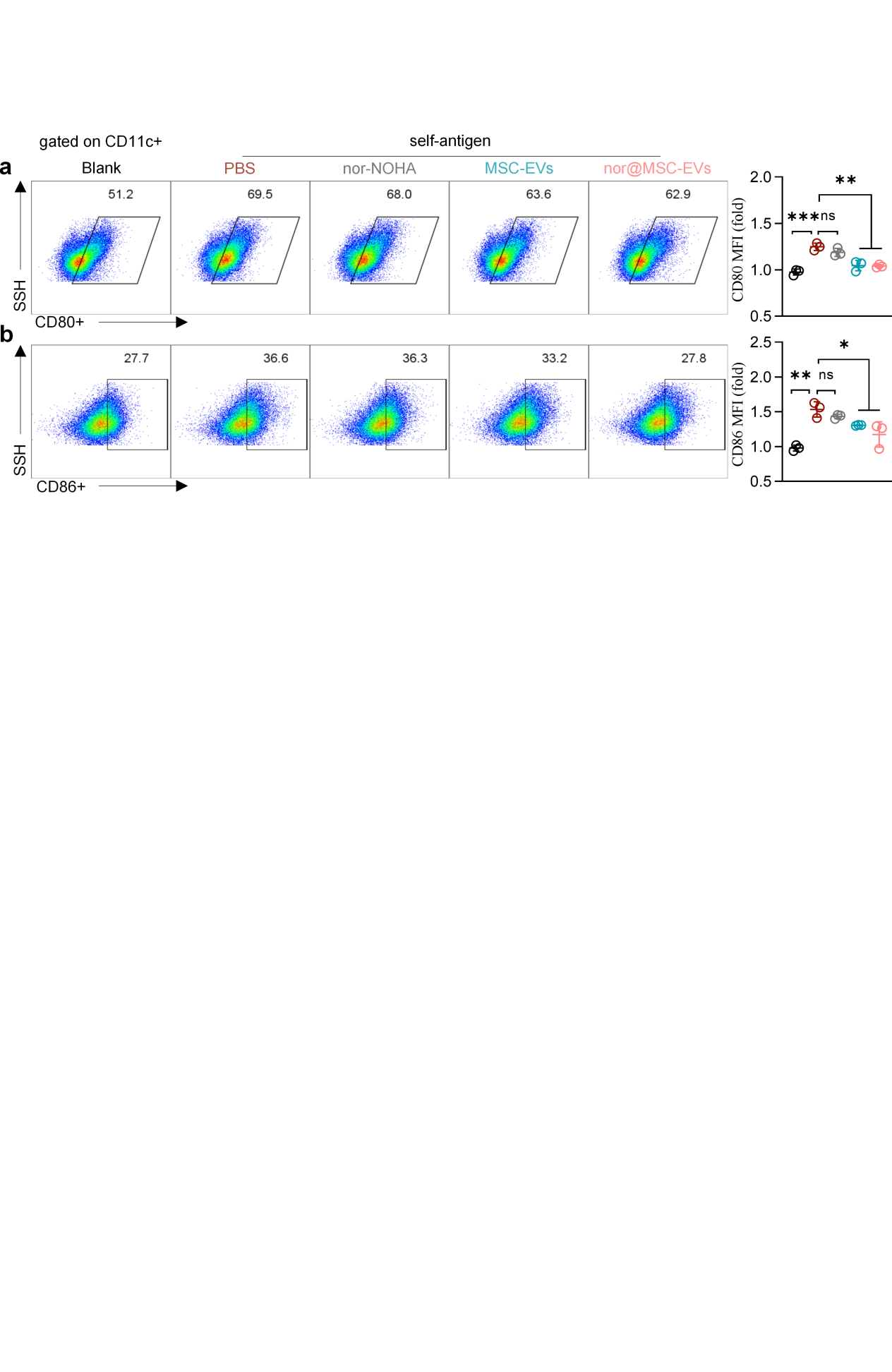


**A**

**B**

**Figure S6.** Nor@MSC-EVs directly modulated immunological disorder *in vitro.* A) and B) The expression level of MFI CD80 (fold) (A) and MFI CD86 (fold) (B) in BMDCs treated by PBS, nor-NOHA, MSC-EVs, or nor@MSC-EVs analyzed by flow cytometry. Data are expressed as mean ± S.D. (n=3). Statistical significance was calculated *via* one-way ANOVA with a Tukey’s test. ns, not significant; ns, not significant; *P<0.05, **P<0.01, ***P<0.001.


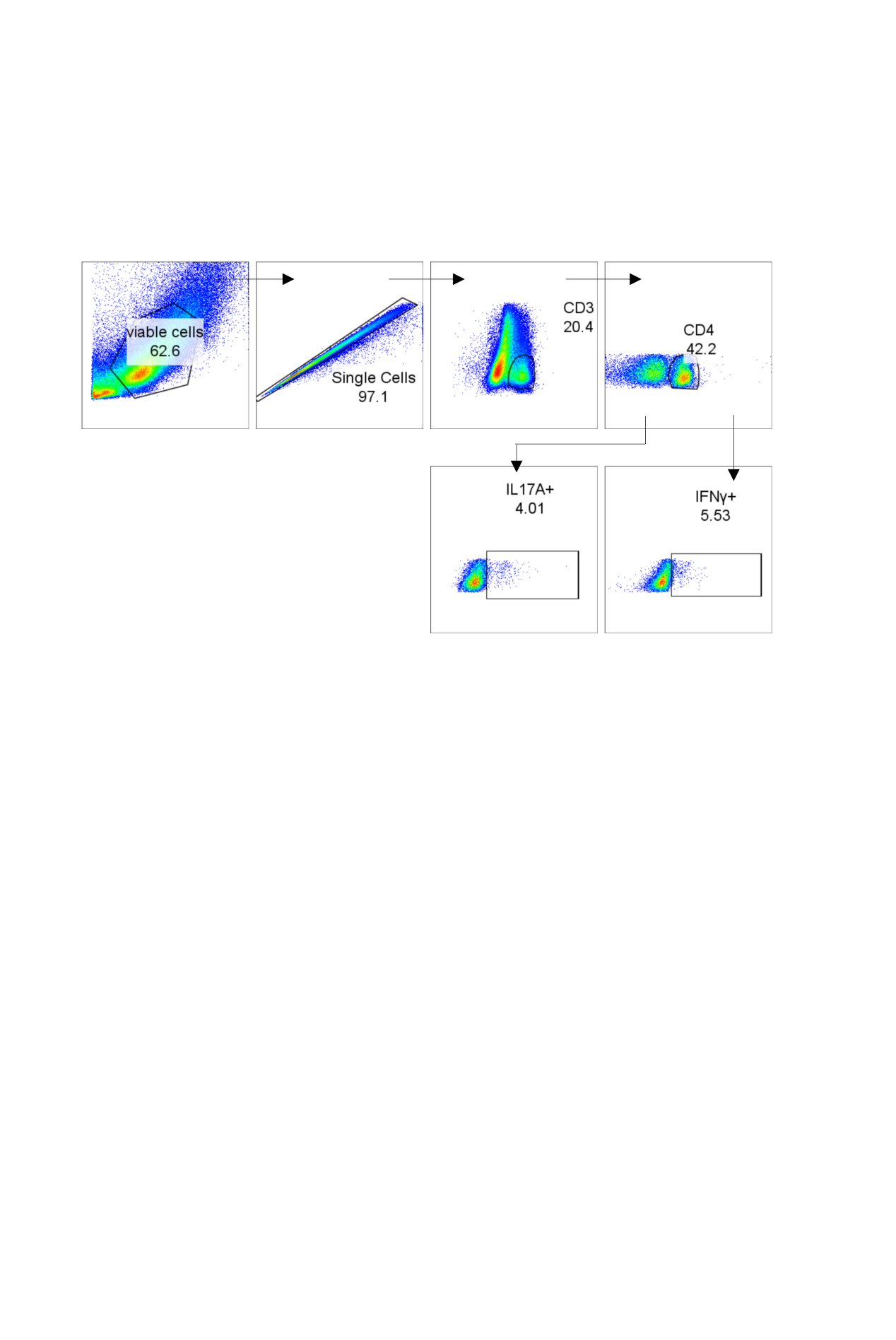


**Figure S7.** Flow cytometry gating strategy of CD4+ T cells *in vitro.*


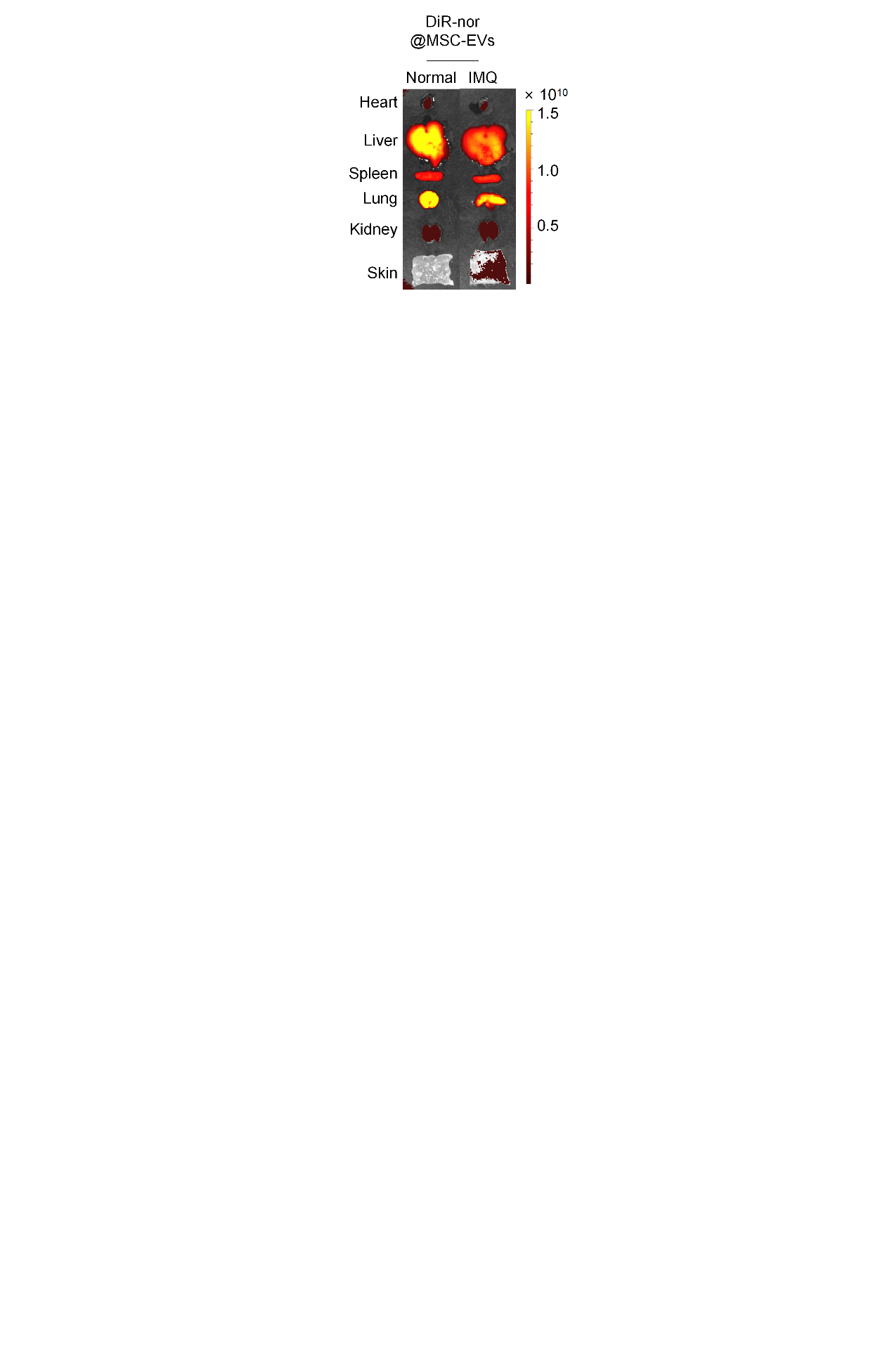


**Figure S8.** The biodistribution of DiR-labeled nor@MSC-EVs in the heart, liver, spleen, lung, kidney, and skin in normal and IMQ mouse.


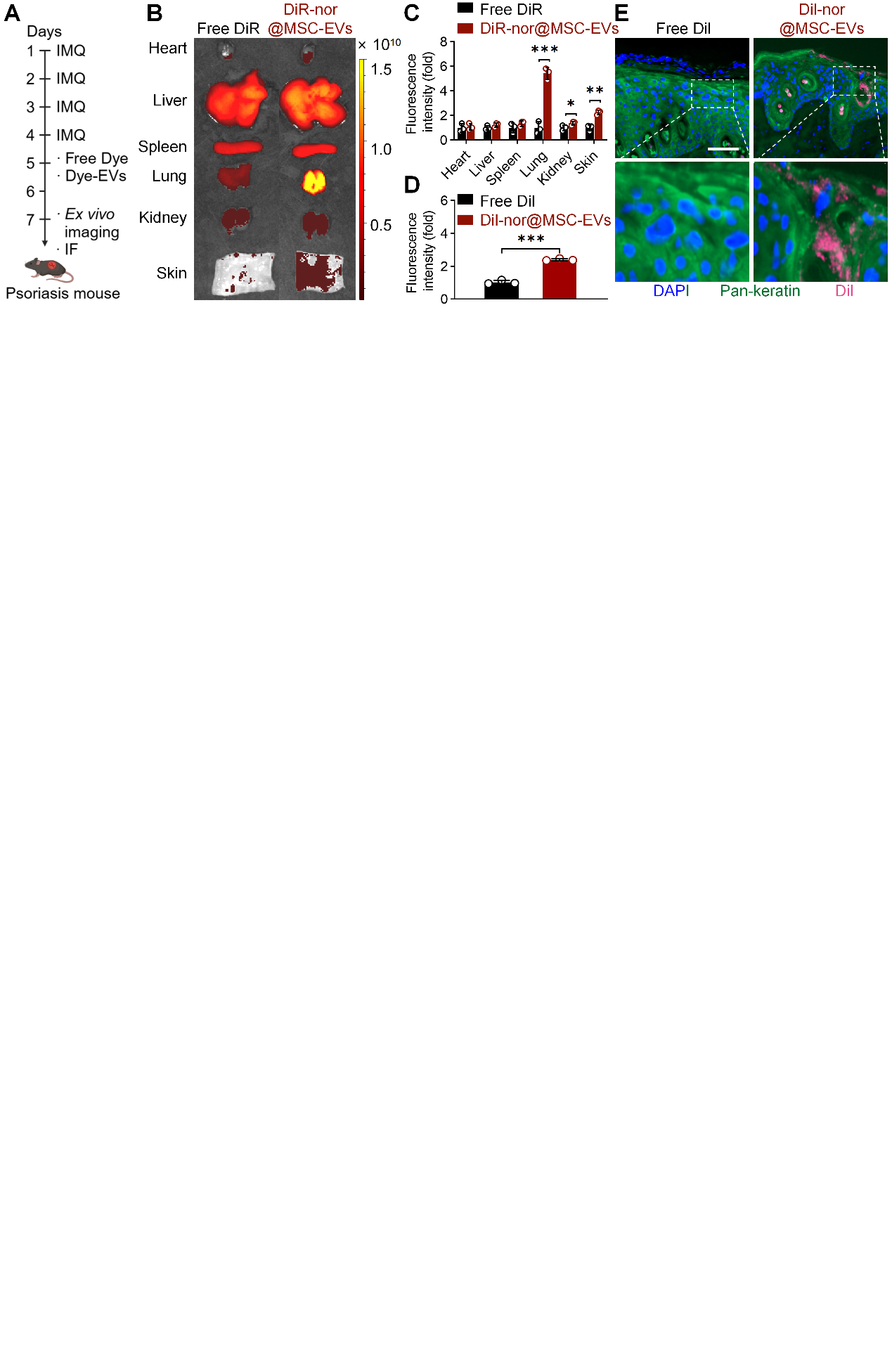


**Figure S9.** Nor@MSC-EVs are taken up by keratinocytes in psoriatic skin lesion. A) Experimental schedule for analyzing the distribution of nor@MSC-EVs in psoriasis mouse. B) After intravenous injection, the biodistribution of Free-DiR and DiR-labeled nor@MSC-EVs in the heart, liver, spleen, lung, kidney, and skin. C) The fluorescence intensity of Free DiR and Dir-nor@MSC-EVs in above organs. D) The fluorescence intensity of Free DiR and DiR-nor@MSC-EVs in keratinocytes. E) Representative immunofluorescence images of Free Dil or Dil-labeled nor@MSC-EVs (pink), pan-keratin positive keratinocytes (green), and DAPI positive cell nucleus (blue). Scale bar=50 μm, n=3. Statistical significance was calculated *via* unpaired two-tailed t test. ns, not significant; ***P<0.001.


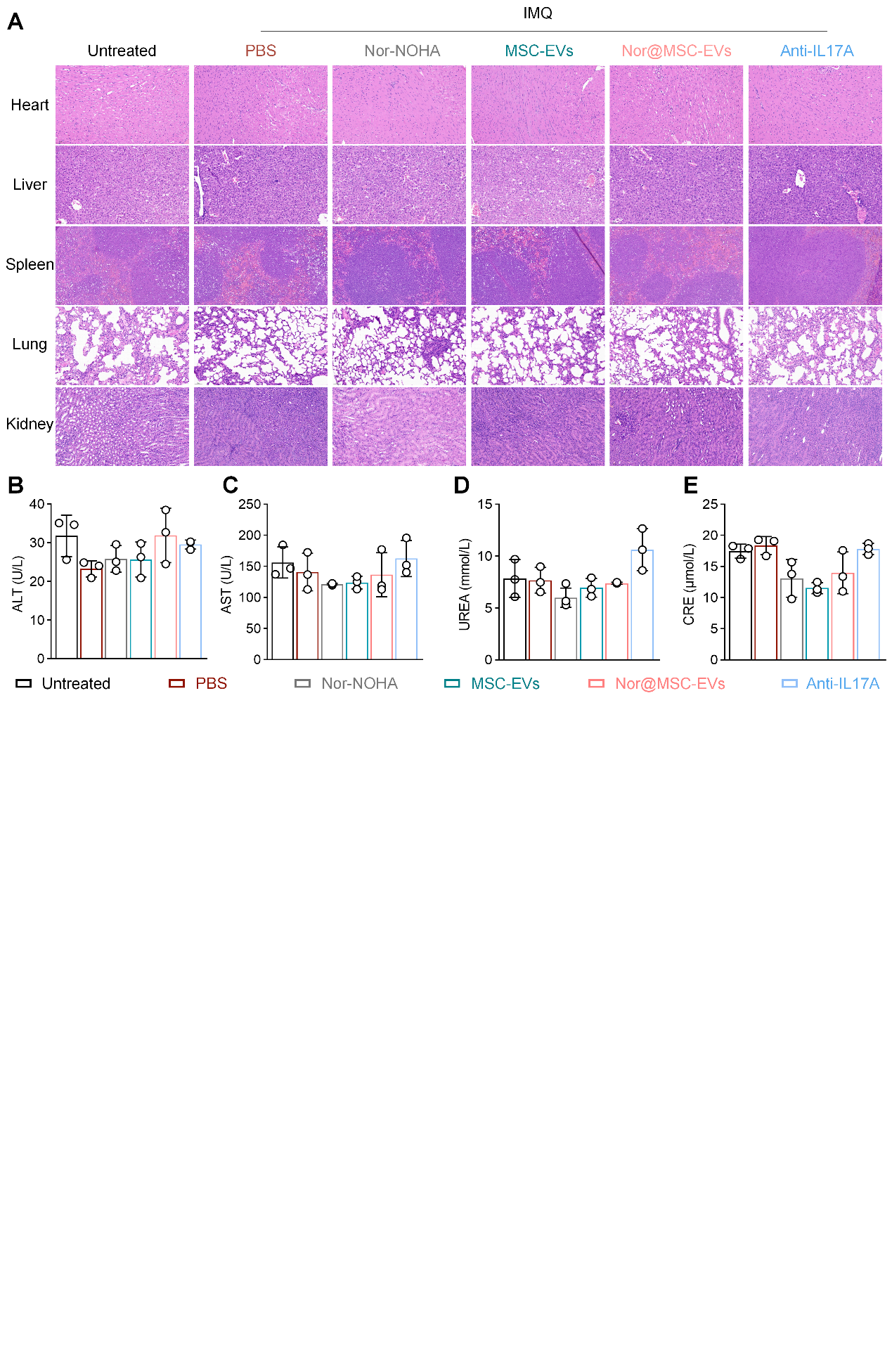


**Figure S10.** Biosafety evaluation of nor@MSC-EVs *in vivo*. **A**) H&E staining of the main organs in different groups after 6 days of treatment to IMQ induced-psoriasis mice. **B**) and **C**) Liver function (ALT and AST), **D**) and **E**) kidney function (UREA and CRE) were evaluated by blood biochemical examination. n=3.


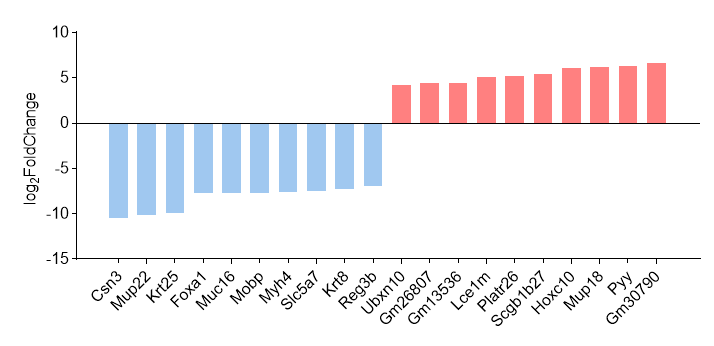


**Figure S11.** The top 10 downregulated and upregulated DEGs in the nor@MSC-EVs group compared to the PBS group.


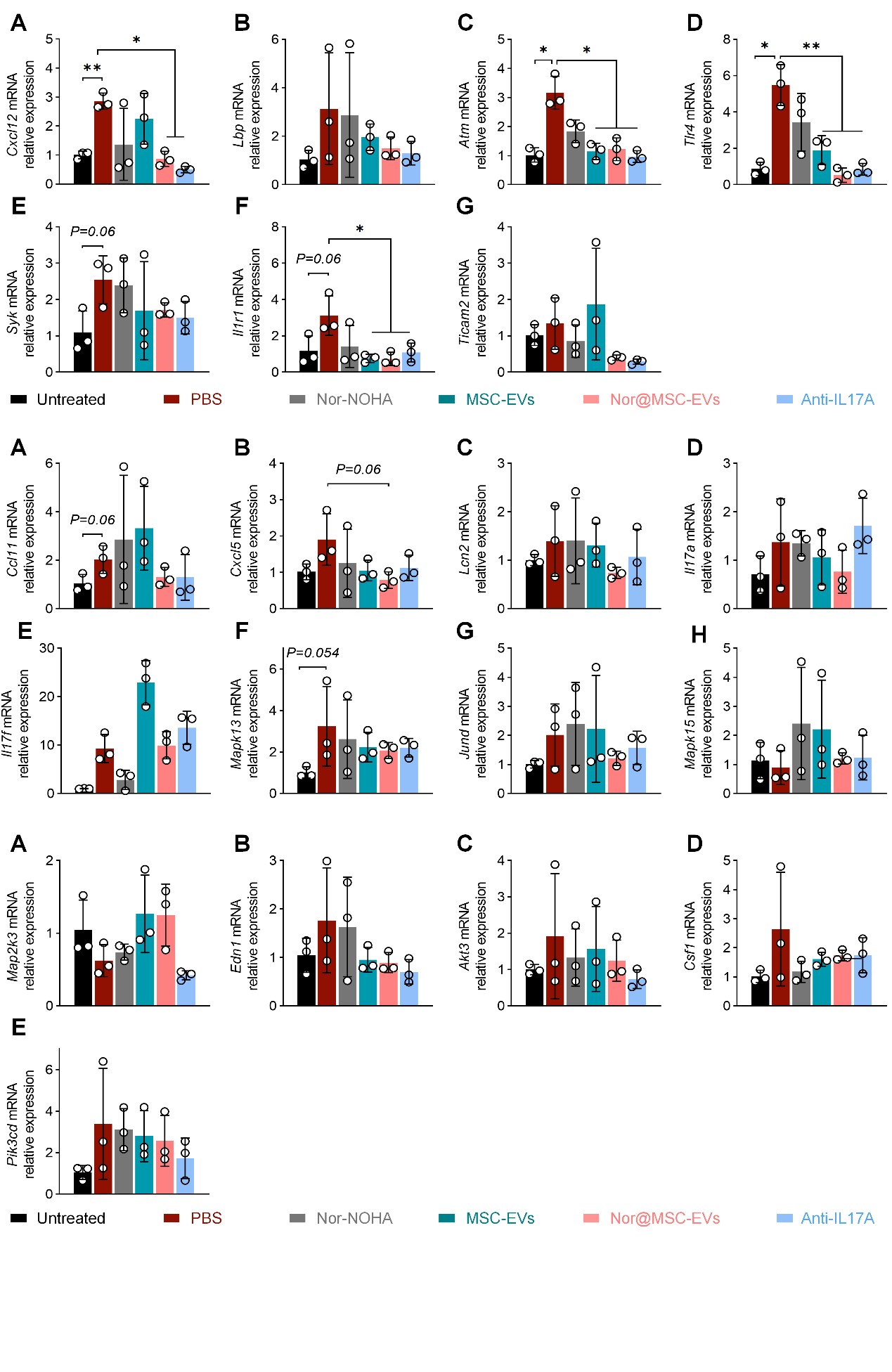


**Figure S12.** Verification of the differentially expressed genes in NF-κB signaling pathway using RT-qPCR. A) *Cxcl12*, B) *Lbp,* C) *Atm*, D) *Tlr4*, E) *Syk*, F) *Il1r1*, G) *Ticam2*. ns, not significant; *P<0.05, **P<0.01, ***P<0.001.

**
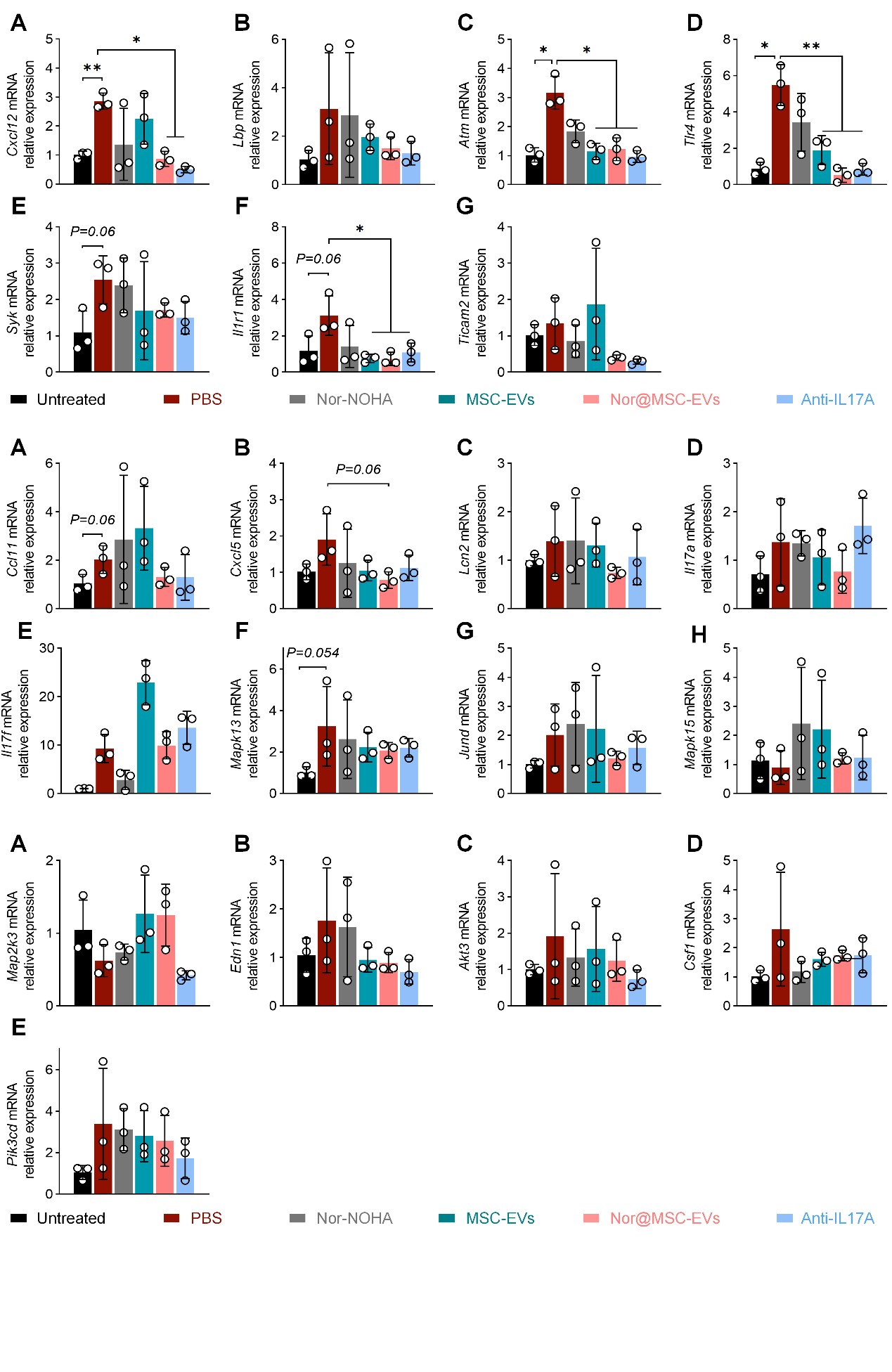
**

**
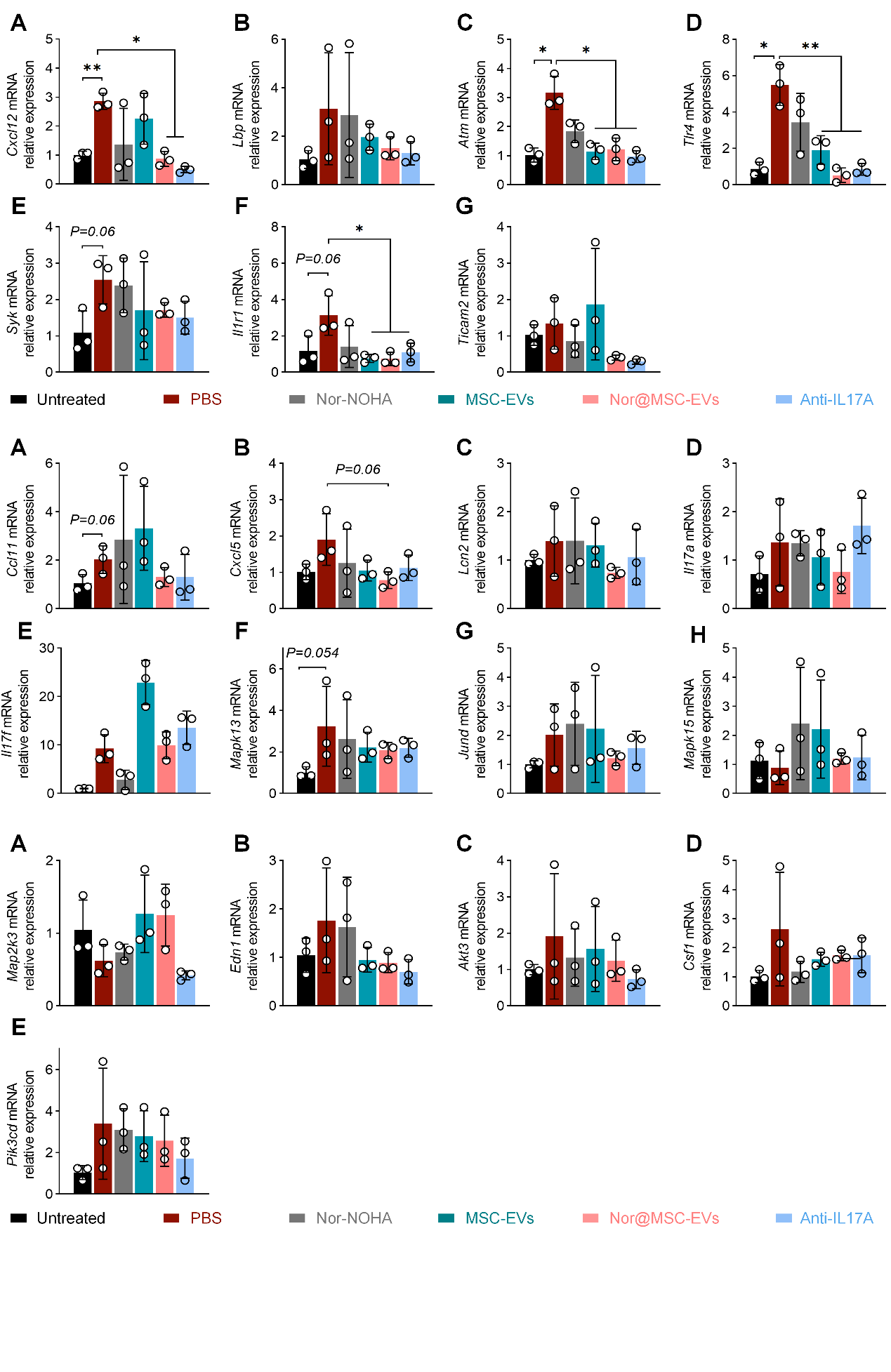
**

**Figure** **S13.** Verification of the differentially expressed genes in IL-17 signaling pathway using RT-qPCR. A) *Ccl11,* B) *Cxcl5,* C) *Lcn2,* D) *Il17a,* E) *Il17f,* F) *Mapk13,* G) *Jund,* H) *Mapk15.* ns, not significant; *P<0.05, **P<0.01, ***P<0.001.

**
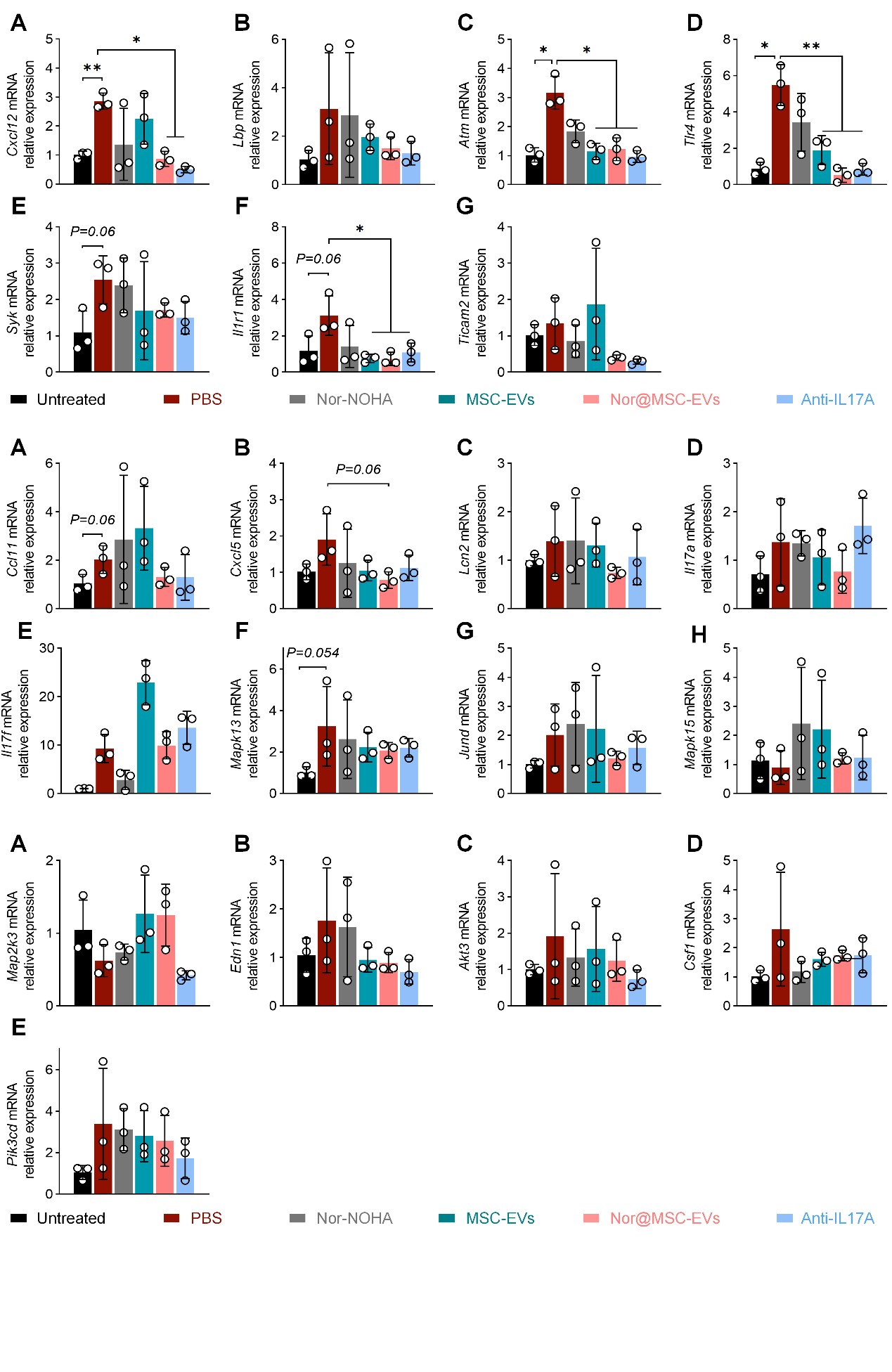
**

**Figure S14.** Verification of the differentially expressed genes in TNF signaling pathway using RT-qPCR. A) *Map2k3*, B) *Edn1*, C) *Akt3*, D) *Csf1*, E) *Pik3cd*. ns, not significant; *P<0.05, **P<0.01, ***P<0.001.


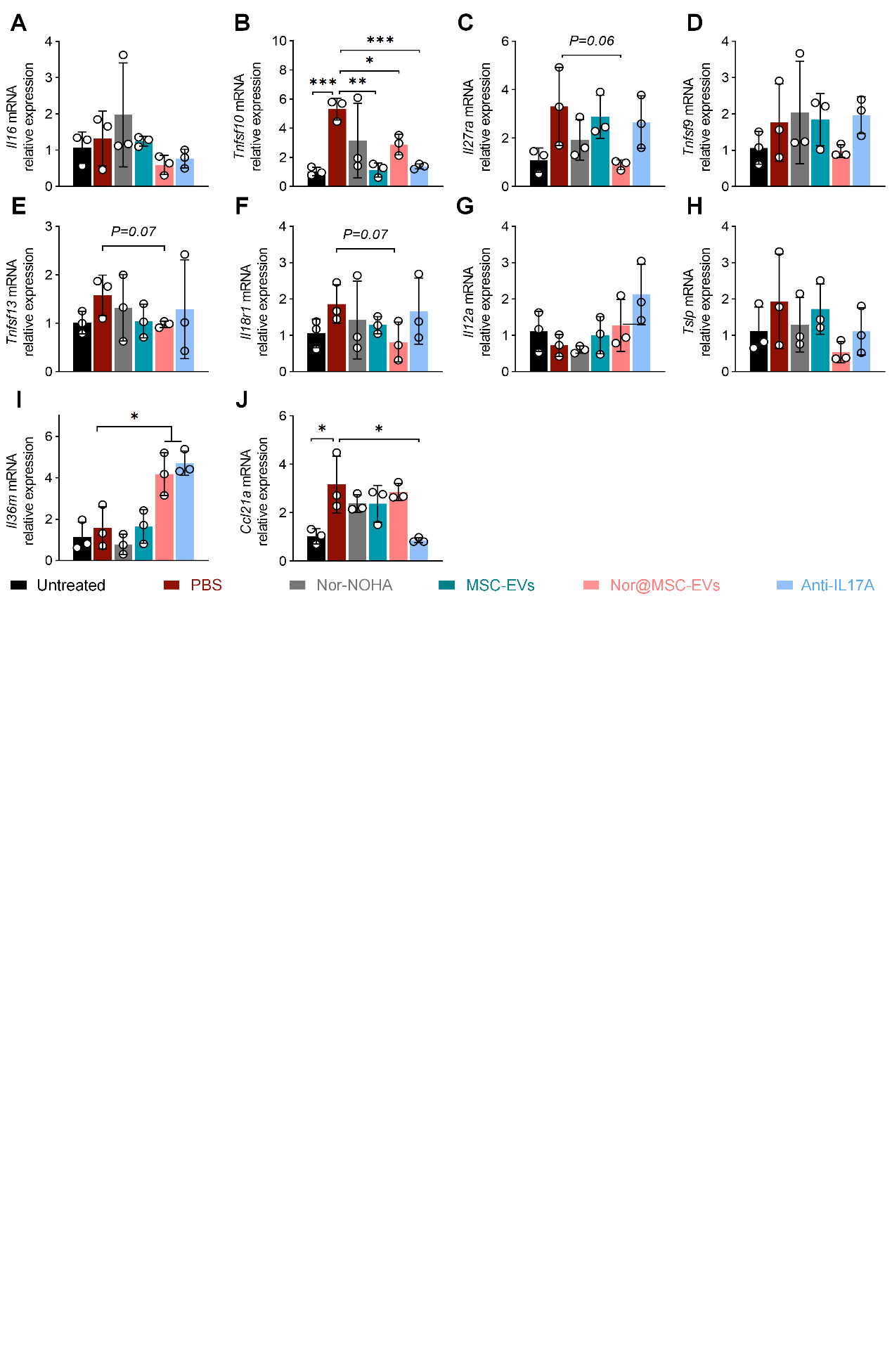


**Figure S15.** Verification of the differentially expressed genes in cytokine-cytokine receptor interaction using RT-qPCR. A) *Il16*, B) *Tnfsf10*, C) *Il27ra*, D) *Tnfsf9*, E) *Tnfsf13*, F) *Il18r1*, G) *Il12a*, H) *Tslp*, I) *Il36m*, J) *Ccl21a*. ns, not significant; *P<0.05, **P<0.01, ***P<0.001.


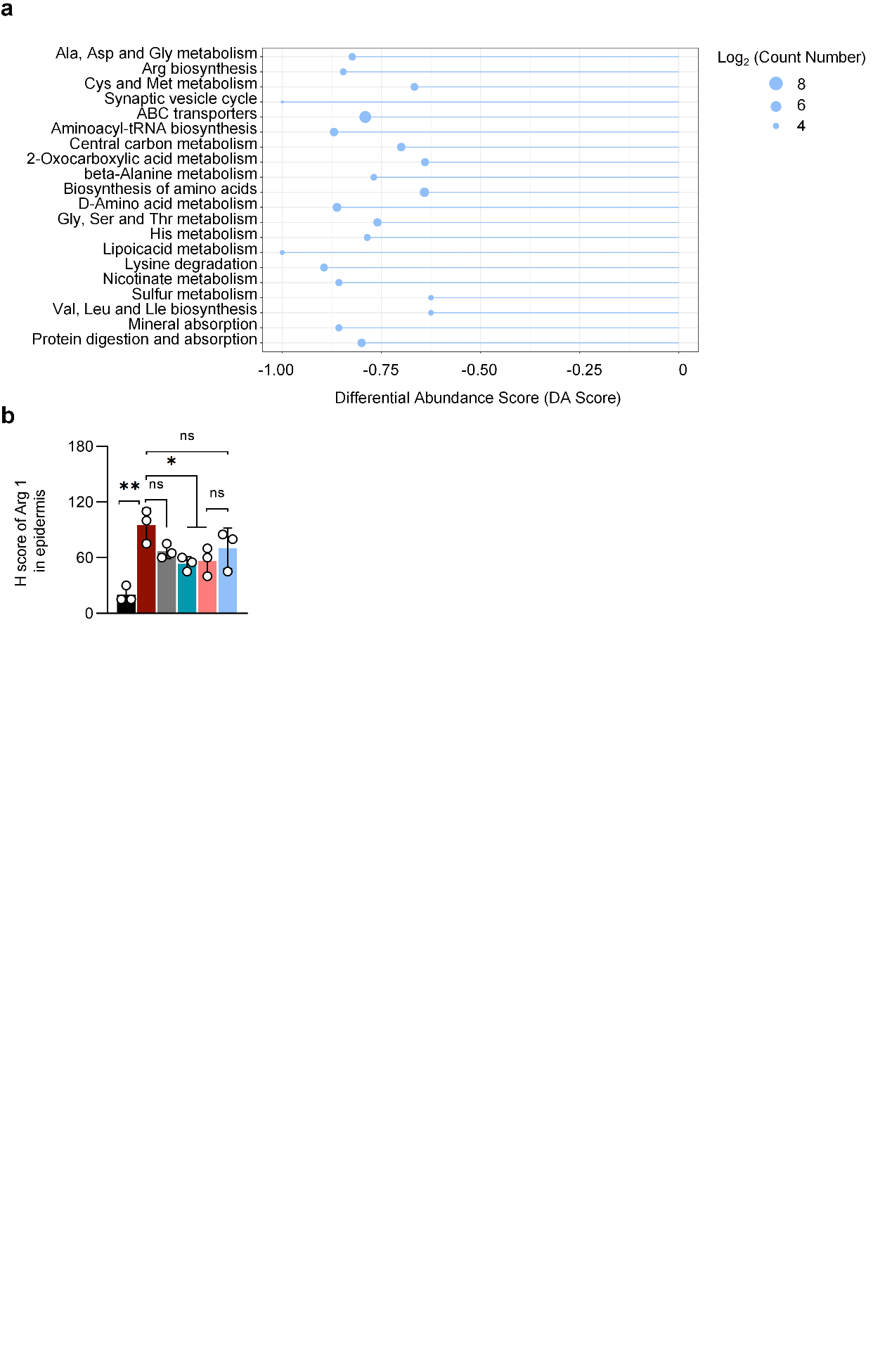


**Figure S16.** Pathway-based analysis of metabolomic changes between the nor@MSC-EVs and PBS groups. The differential abundance score (DA score) represents the average, overall changes for all metabolites within a pathway. A DA score greater than 0 indicates an increased trend in the pathway in the nor@MSC-EVs group compared to the PBS group, while a DA score less than 0 indicates a decreased trend in the pathway in the nor@MSC-EVs group compared to the PBS group.


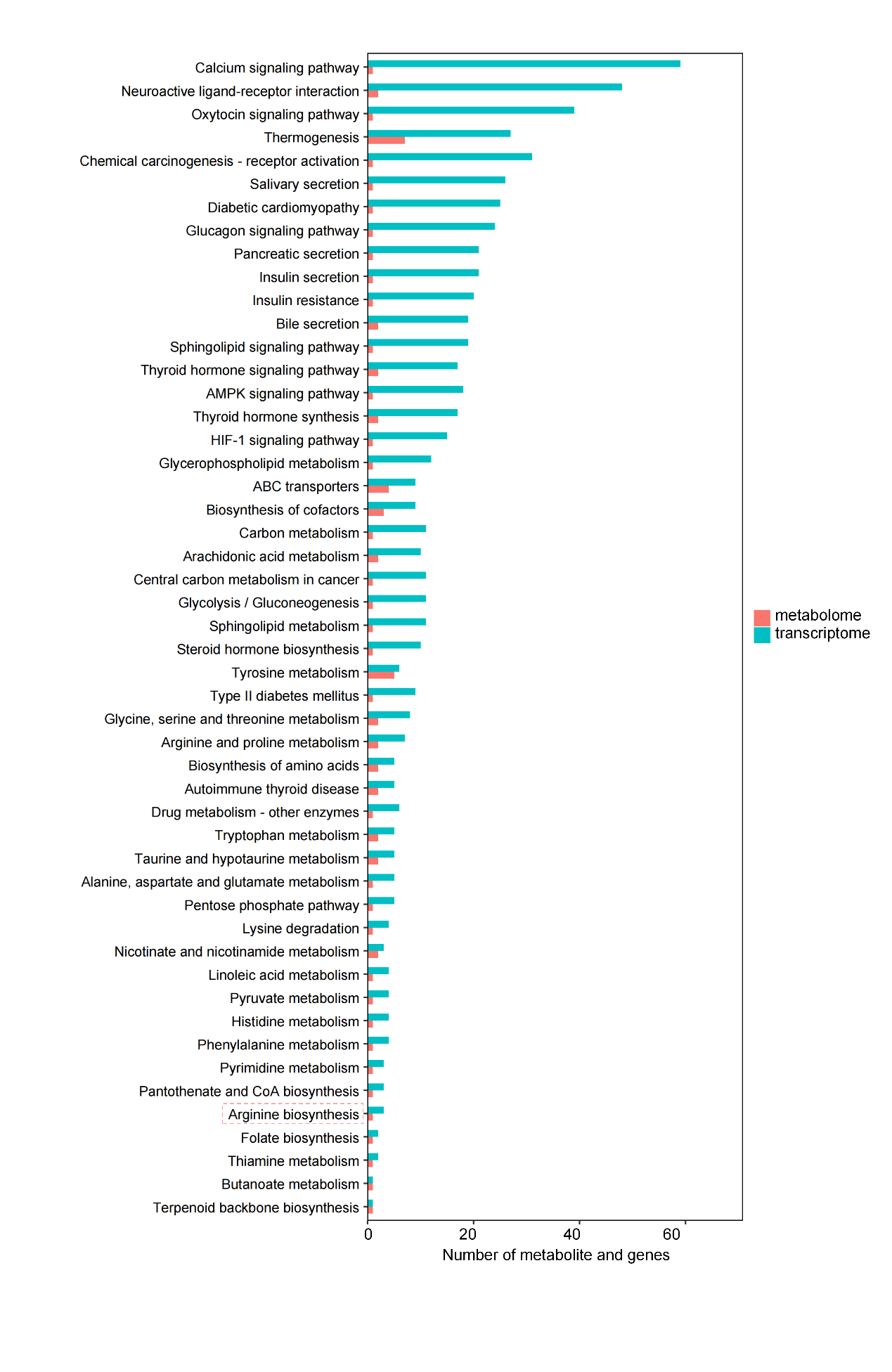
**Figure S17.** Conjoint analysis of metabolome and transcriptome in the nor@MSC-EVs group compared to the PBS group.


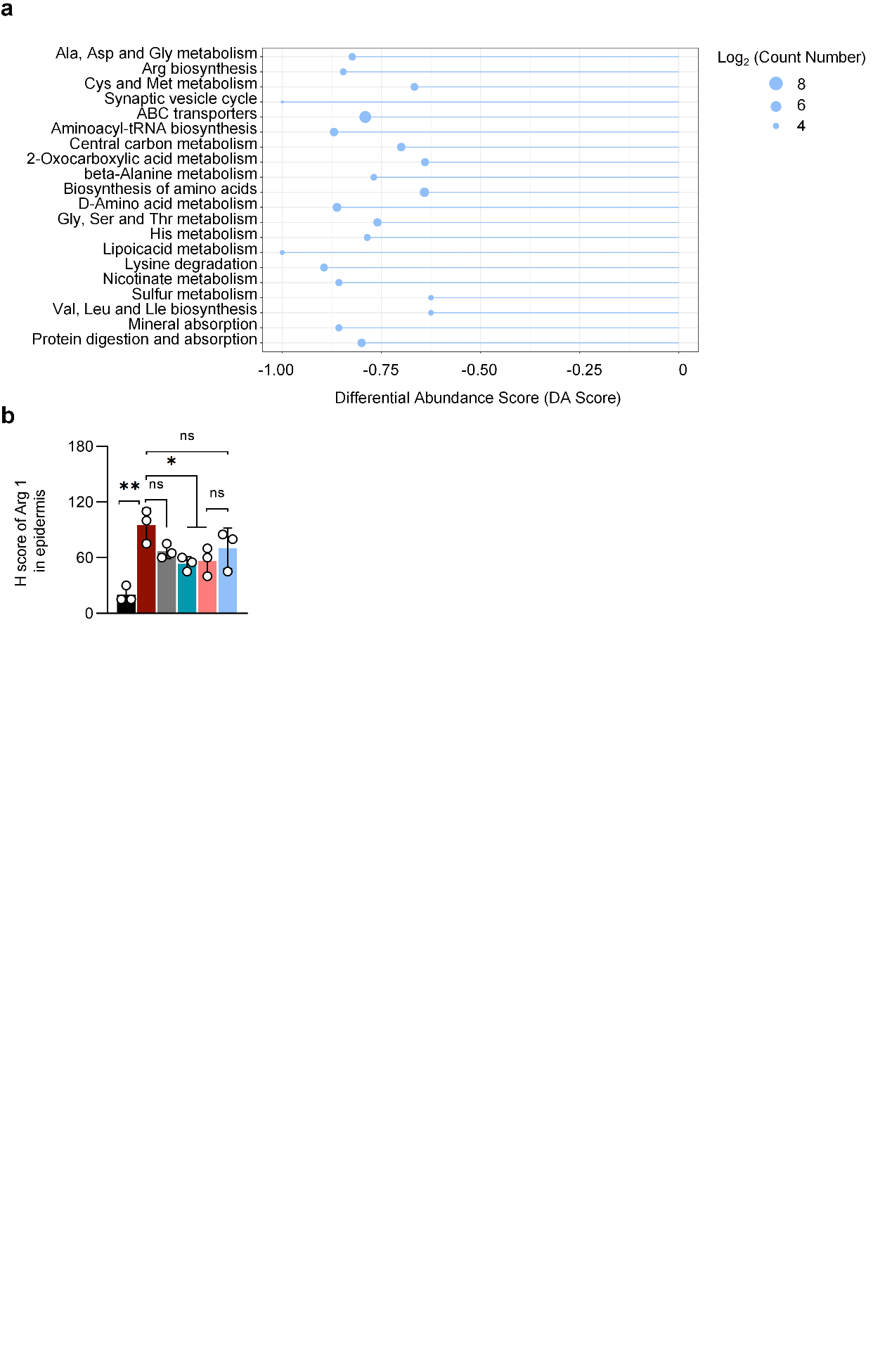


**Figure S18.** Quantification of the H score of Arg1+ in the epidermis. Data are expressed as mean ± S.D. (n=3). Statistical significance was calculated *via* one-way ANOVA with a Tukey’s test. ns, not significant; *P<0.05, **P<0.01.


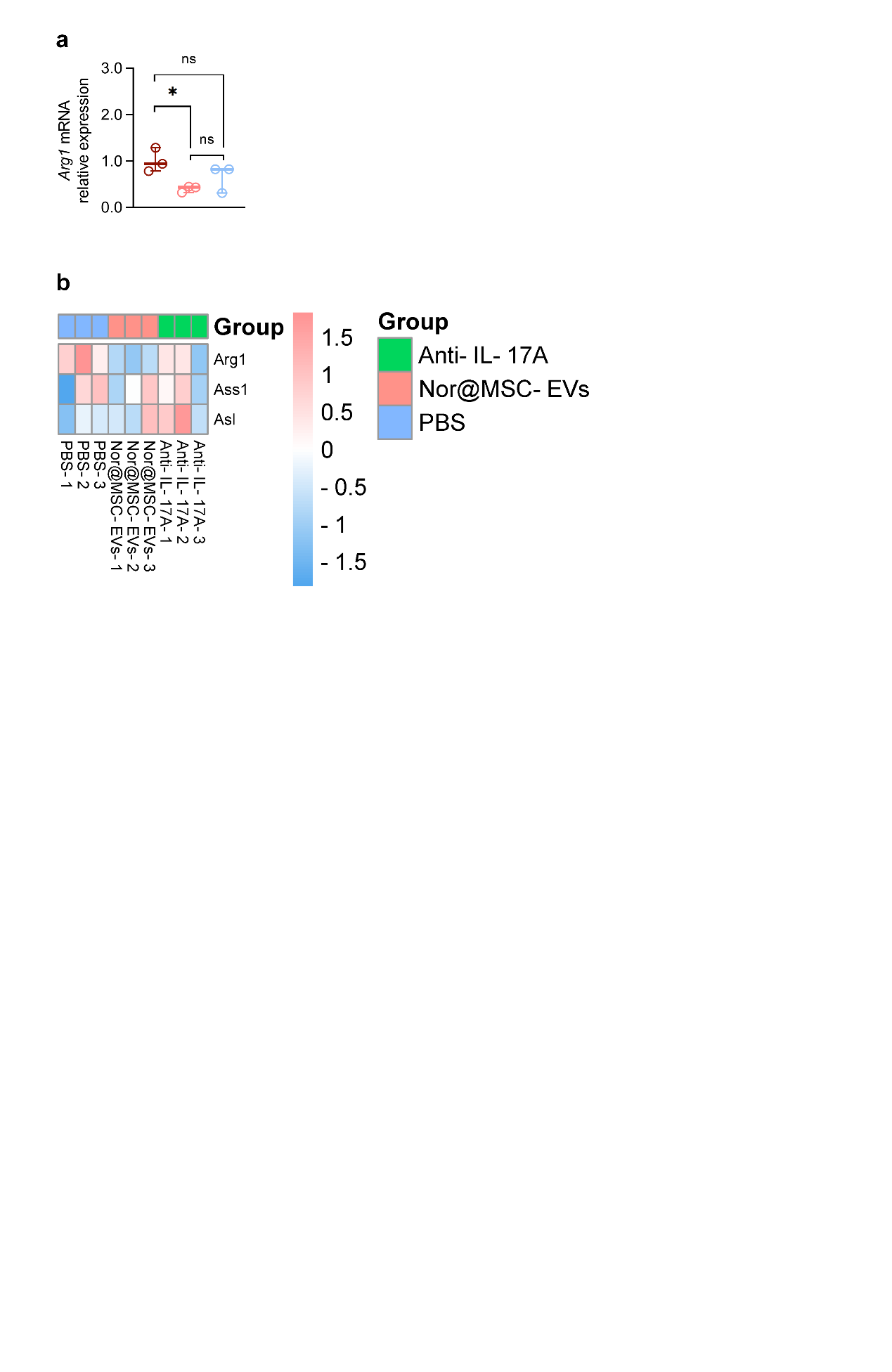


**Figure S19**. Relative expression of Arg1 *via* RNA-seq. Data are expressed as mean ± S.D. (n=3). Statistical significance was calculated *via* one-way ANOVA with a Tukey’s test. ns, not significant; *P<0.05.


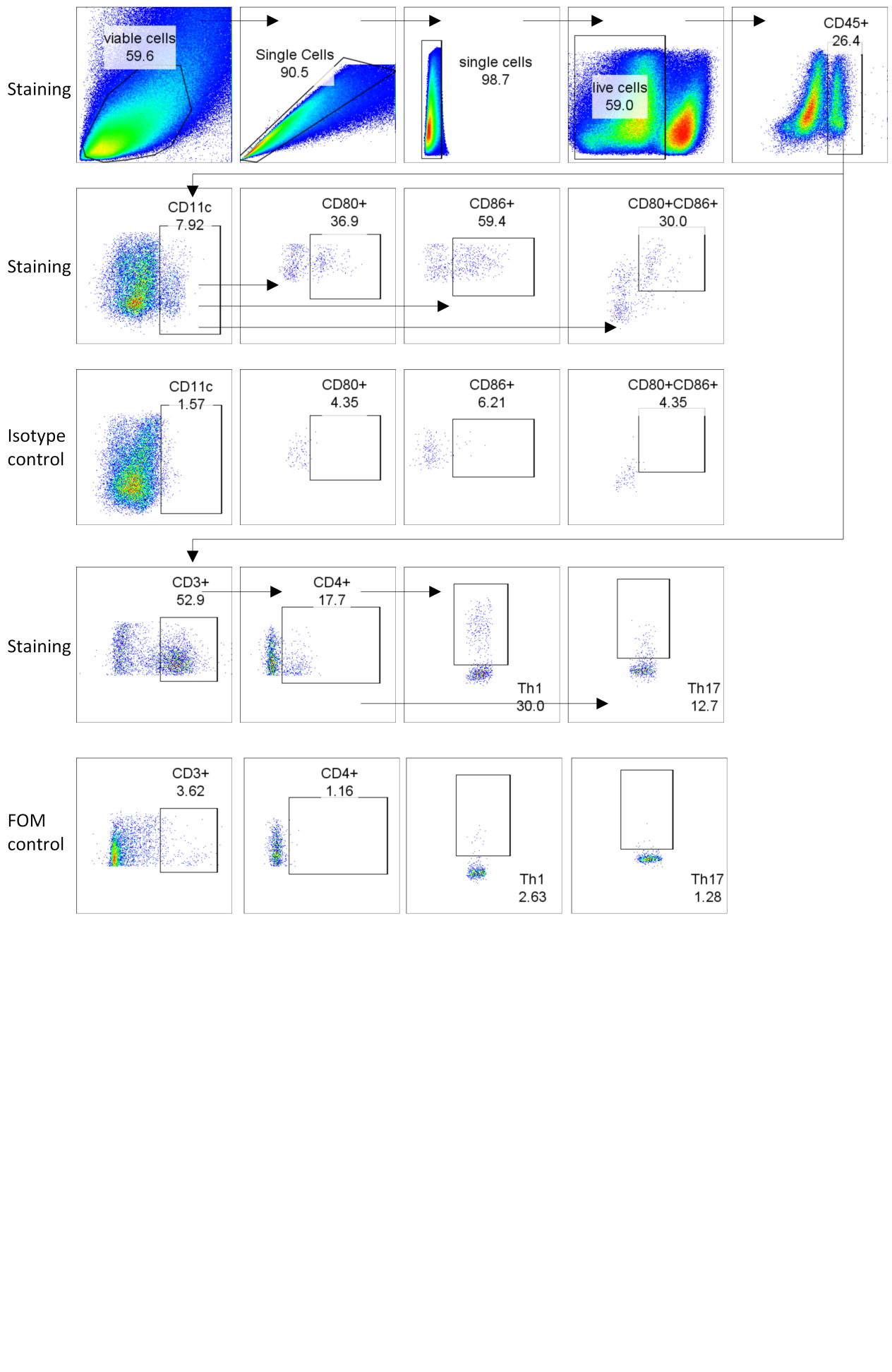


**Figure S20.** Flow cytometry gating strategy and isotype/FMO control for analyzing mature DCs and Th1/17 cells in skin.


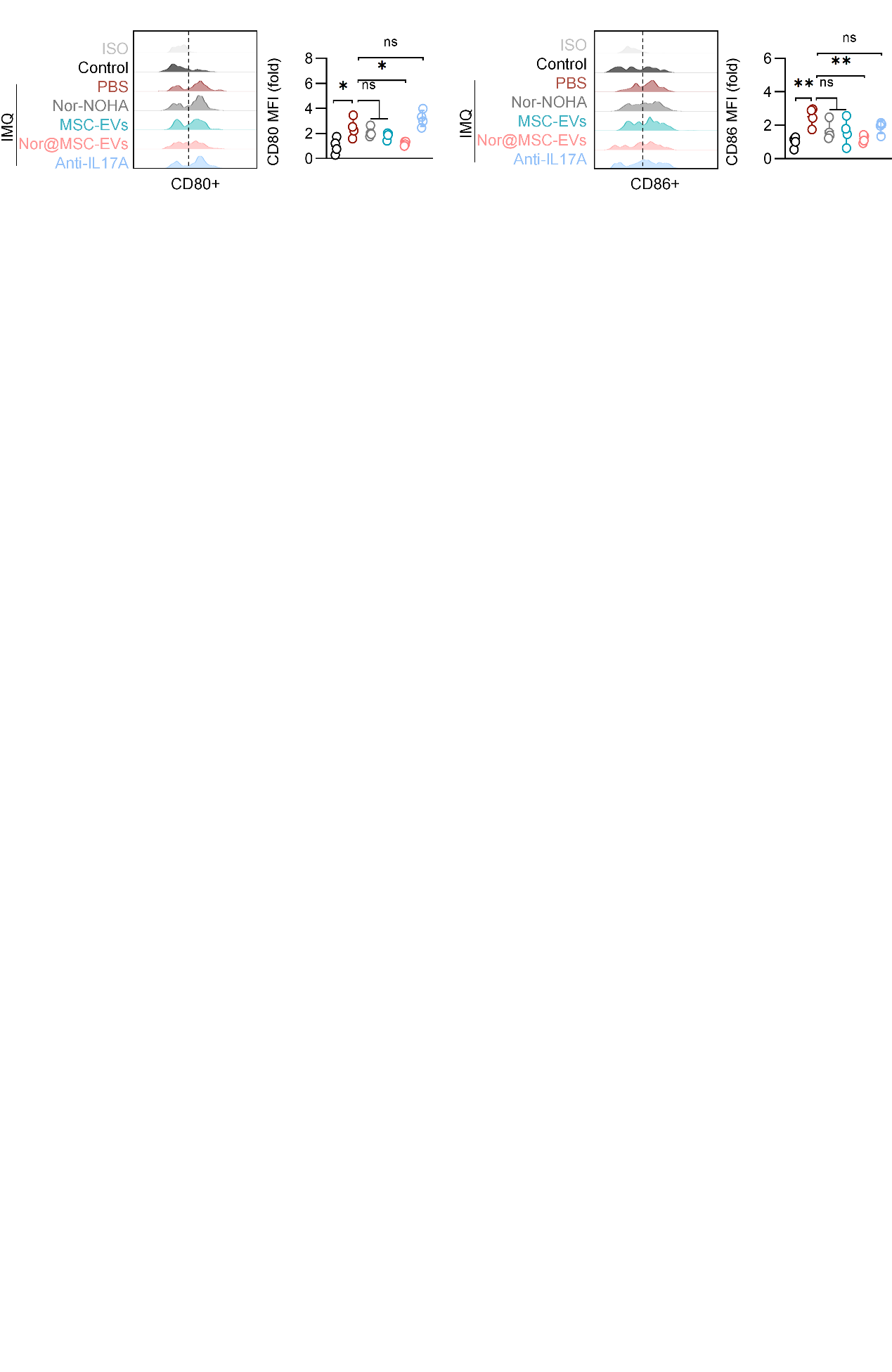


**Figure S21.** CD80 and CD86 MFI of DCs in skin. Data are expressed as mean ± S.D. (n=3). Statistical significance was calculated *via* one-way ANOVA with a Tukey’s test. ns, not significant; *P<0.05, **P<0.01.


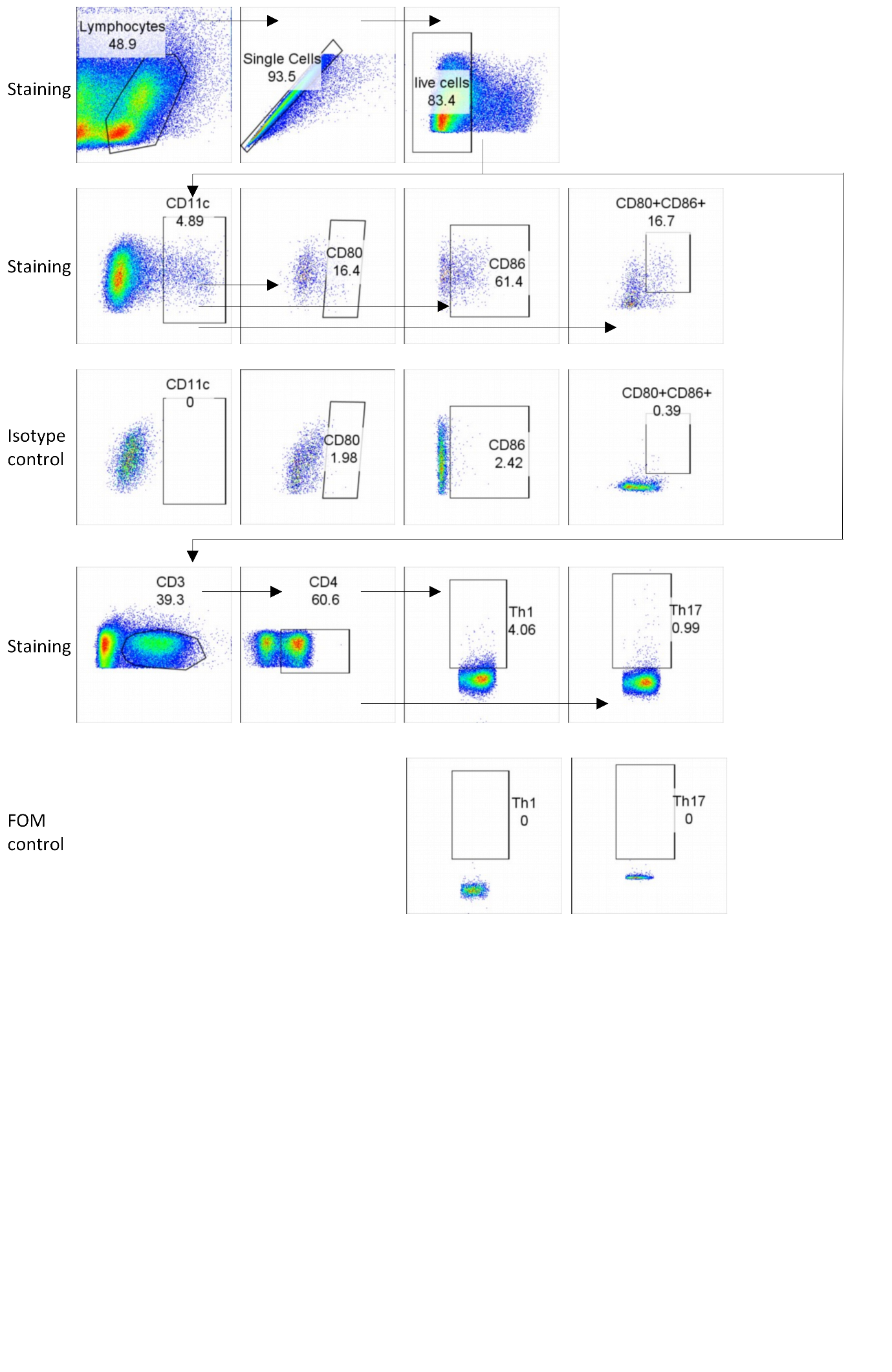


**Figure S22.** Flow cytometry gating strategy and isotype/FMO control for analyzing mature DCs and Th1/17 cells in spleen and lymph node.


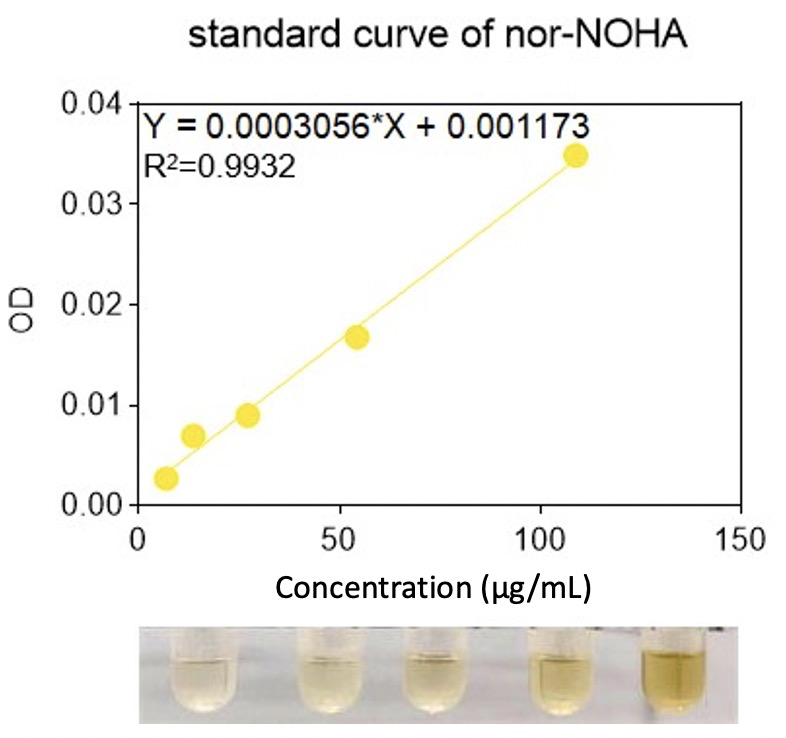


**Figure S23.** Standard curve of nor-NOHA.

**Supplementary Table**

**Table S1.** Encapsulation efficiency, drug loading efficiency, and drug loading per nor@MSC-EVs.


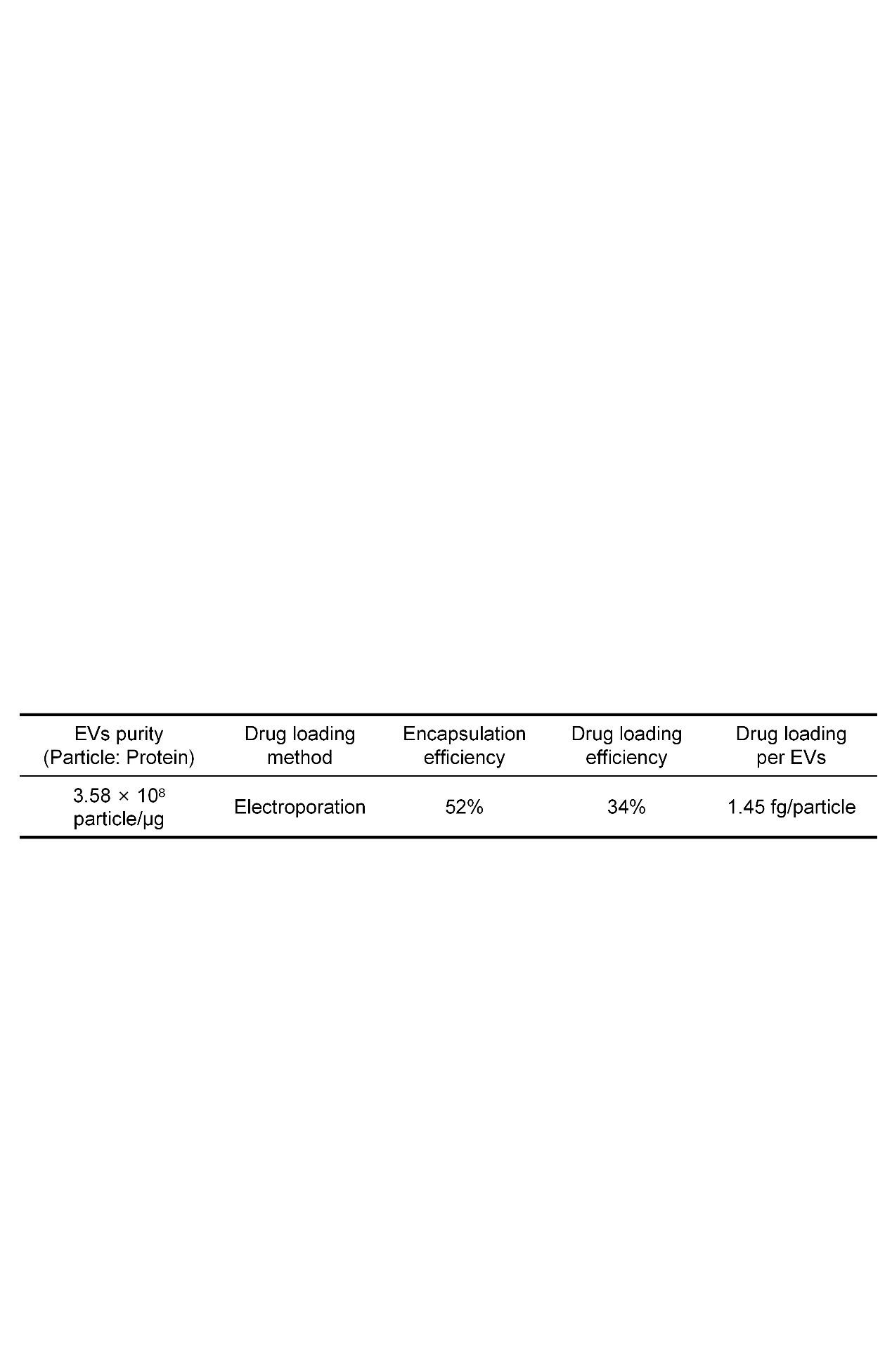


**Table S2.** Primers for analyzing gene expression in HaCaT.

| Primer | Sequence |
| --- | --- |
| GAPDH-F | GTCTCCTCTGACTTCAACAGCG |
| GAPDH-R | ACCACCCTGTTGCTGTAGCCAA |
| S100A8-F | ATGCCGTCTACAGGGATGACCT |
| S100A8-R | AGAATGAGGAACTCCTGGAAGTTA |
| S100A9-F | GCACCCAGACACCCTGAACCA |
| S100A9-R | TGTGTCCAGGTCCTCCATGATG |
| CAMP-F | GACACAGCAGTCACCAGAGGAT |
| CAMP-R | TCACAACTGATGTCAAAGGAGCC |
| DEFB4A-F | ATAGGCGATCCTGTTACCTGCC |
| DEFB4A-R | CATCAGCCACAGCAGCTTCTTG |
| IL1B-F | CCACAGACCTTCCAGGAGAATG |
| IL1B-R | GTGCAGTTCAGTGATCGTACAGG |
| TNFA-F | CTCTTCTGCCTGCTGCACTTTG |
| TNFA-R | ATGGGCTACAGGCTTGTCACTC |
| CCL2-F | AGAATCACCAGCAGCAAGTGTCC |
| CCL2-R | TCCTGAACCCACTTCTGCTTGG |
| CCL20-F | AAGTTGTCTGTGTGCGCAAATCC |
| CCL20-R | CCATTCCAGAAAAGCCACAGTTTT |
| PPP6C-F | CCGCTGGATCTGGACAAGTAT |
| PPP6C-R | ACACTGGCTGAACATTCGACT |
| Arg1-F | CCCTGGGGAACACTACATTTTG |
| Arg1-R | GCCAATTCCTAGTCTGTCCACTT |

**Table S3.** Primers for analyzing gene expression in mouse.

| Primer | Sequence |
| --- | --- |
| Cxcl12-F | GGAGGATAGATGTGCTCTGGAAC |
| Lbp-F | TCCATCGGTGTCCGAGGCAAAT |
| Atm-F | CCAAGATGGCAGTGAACCAGAC |
| Tlr4-F | AGCTTCTCCAATTTTTCAGAACTTC |
| Syk-F | GAGAGCACTGTGTCCTTCAACC |
| Il1r1-F | CTGTTGGTGAGGAATGTGGCTG |
| Tirap-F | ATCTCCCAGGAAAGCCACCTCT |
| Ticam2-F | ATCAGAGCCACCAACAGGAGAG |
| Ccl11-F | GAATCACCAACAACAGATGCAC |
| Cxcl5-F | CCGCTGGCATTTCTGTTGCTGT |
| Lcn2-F | ATGTCACCTCCATCCTGGTCAG |
| Il17a-F | CAGACTACCTCAACCGTTCCAC |
| Il17f-F | AACCAGGGCATTTCTGTCCCAC |
| Mapk13-F | CAGCGAGGATAAGGTCCAGTAC |
| Jund-F | ACCTGCACAAGCAAAGCCAGCT |
| Mapk15-F | TCCAGGACCTTGGCTCAGACTA |
| Map2k3-F | GGACCTTCATCACTATCGGAGAC |
| Edn1-F | CTACTTCTGCCACCTGGACATC |
| Akt3-F | GAGATGGATGCGTCTACAACCC |
| Csf1-F | GCCTCCTGTTCTACAAGTGGAAG |
| Pik3cd-F | ACCATCAGTGGCTCTGCGGTTT |
| Il16-F | CACGCAGACTTCATCCTCCACA |
| Tnfsf10-F | GGAAGACCTCAGAAAGTGGCAG |
| Il27ra-F | CCAACCTGTCTCTGGTGTGCTT |
| Tnfsf9-F | CCCTGTTTCCCACATTGGCTGC |
| Tnfsf13-F | TCTCGGAGAAGGAGAGCAGTAC |
| Il18r1-F | AGAGCTGATCCAGGACACATGG |
| Il12a-F | ACGAGAGTTGCCTGGCTACTAG |
| Tslp-F | GCAAATCGAGGACTGTGAGAGC |
| Il36rn-F | CAAGGAGGAAGCCAGTGCCTAT |
| Ccl21a-F | GGGTCAGGACTGCTGCCTTAAG |
| Cxcl12-R | AGTGAGGATGGAGACCGTGGTG |
| Lbp-R | AGGTCCACTGAAATGGTGACACC |
| Atm-R | ATGCTGGACAGCTATGGTGGAG |
| Tlr4-R | TGAGAGGTGGTGTAAGCCATGC |
| Syk-R | CAGCATAAGGGCTCTCGTACAC |
| Il1r1-R | GGCTCAGGATAACAGGTCTGTC |
| Tirap-R | GGTAGGTGACATTCCTGAACTGC |
| Ticam2-R | CTCGTCGGTGTCATCTTCTGCA |
| Ccl11-R | CTCTTTGCCCAACCTGGTCTTG |
| Cxcl5-R | CAGGGATCACCTCCAAATTAGCG |
| Lcn2-R | GCCACTTGCACATTGTAGCTCTG |
| Il17a-R | TCCAGCTTTCCCTCCGCATTGA |
| Il17f-R | GGCATTGATGCAGCCTGAGTGT |
| Mapk13-R | GCTCACAGTCTTCATTCACAGCC |
| Jund-R | CGAAACTGCTCAGGTTGGCGTA |
| Mapk15-R | AGCAAACGCCAAGAGTCGCTTG |
| Map2k3-R | ATGCCGCACTTTCTCTACCACC |
| Edn1-R | CGCACTGACATCTAACTGCCTG |
| Akt3-R | TCCACTTGCCTTCTCTCGAACC |
| Csf1-R | ACTGGCAGTTCCACCTGTCTGT |
| Pik3cd-R | GTGGTCTTCTGGGAACTCACCT |
| Il16-R | AGCTATAGTCCATCCGTGCCTG |
| Tnfsf10-R | TTTCCGAGAGGACTCCCAGGAT |
| Il27ra-R | TACTCCAACGGTTTCCTGGTCC |
| Tnfsf9-R | CTCCATCTTGGCTGTGCCAGTT |
| Tnfsf13-R | ACTGGTTGCCACATCACCTCTG |
| Il18r1-R | TGGTGGACAGAAAACACGCAGG |
| Il12a-R | CCTCATAGATGCTACCAAGGCAC |
| Tslp-R | TGAGGGCTTCTCTTGTTCTCCG |
| Il36rn-R | TCGAAGCTGGAGGTAAGACCCA |
| Ccl21a-R | AGCTCAGGCTTAGAGTGCTTCC |
